# miRNA-1-3p is an early embryonic male sex-determining factor in the Oriental fruit fly *Bactrocera dorsalis*

**DOI:** 10.1038/s41467-020-14622-4

**Published:** 2020-02-18

**Authors:** Wei Peng, Shuning Yu, Alfred M. Handler, Zhijian Tu, Giuseppe Saccone, Zhiyong Xi, Hongyu Zhang

**Affiliations:** 1 Key Laboratory of Horticultural Plant Biology (MOE) and State Key Laboratory of Agricultural Microbiology, College of Plant Science and Technology, Huazhong Agricultural University, Wuhan, Hubei 430070 People’s Republic of China; 2 China-Australia Joint Research Centre for Horticultural and Urban Pests, Huazhong Agricultural University, Wuhan, Hubei 430070 People’s Republic of China; 3 USDA/ARS, Center for Medical, Agricultural and Veterinary Entomology, 1700 SW 23rd Drive, Gainesville, FL 32608 USA; 4 Department of Biochemistry, Virginia Polytechnic Institute and State University, Blacksburg, VA 24061 USA; 5 Department of Biology, University Federico II of Naples, Via Cinthia 26, Naples, 80126 Italy; 6 Department of Microbiology and Molecular Genetics, Michigan State University, East Lansing, MI 48824 USA

**Keywords:** Agricultural genetics, Non-coding RNAs

## Abstract

Regulation of male sexual differentiation by a Y chromosome-linked male determining factor (M-factor) is one of a diverse array of sex determination mechanisms found in insects. By deep sequencing of small RNAs from *Bactrocera dorsalis* early embryos, we identified an autosomal-derived microRNA, miR-1-3p, that has predicted target sites in the *transformer* gene (*Bdtra*) required for female sex determination. We further demonstrate by both in vitro and in vivo tests that miR-1-3p suppresses *Bdtra* expression. Injection of a miR-1-3p mimic in early embryos results in 87–92% phenotypic males, whereas knockdown of miR-1-3p by an inhibitor results in 67–77% phenotypic females. Finally, CRISPR/Cas9-mediated knockout of miR-1-3p results in the expression of female-specific splice variants of *Bdtra* and *doublesex* (*Bddsx*), and induced sex reversal of XY individuals into phenotypic females. These results indicate that miR-1-3p is required for male sex determination in early embryogenesis in *B. dorsalis* as an intermediate male determiner.

## Abstract (web summary)

In insects, Y chromosome-linked male determining factor can determine sex, but the sex determination factor of the Oriental Fruit fly is unknown. Here the authors show that an autosomal-derived miR-1-3p is enriched in males, suppressing the expression of *Bdtra*, which is required for female sex determination.

## Introduction

Primary signals that initiate sex determination during development appear to be highly divergent among many taxonomic groups^1–3^. This is exemplified in dipteran insects, such as *Drosophila melanogaster*, in which the primary sex-determining signal consists of the dose of X chromosome-linked signal elements (XSE)^4^. A double XSE dose from XX diploid individuals leads to expression of *Sex-lethal* (*Sxl*), which directs female-specific splicing of *transformer* (*tra*) and *doublesex* (*dsx*) pre-mRNA whose gene products sequentially direct female differentiation. In the absence of *Sxl* expression from a single XSE dose in XY (or XO) individuals, male-specific *tra* pre-mRNA splicing results in nonfunctional TRA and the subsequent default mode of *dsx* pre-mRNA male-specific splicing, with a resulting DSX-male product that directs male differentiation^5–7^. Although the *Drosophila* Y chromosome is necessary for male fertility, it has no apparent role in determining somatic and germline sexual differentiation^8,9^. This is in contrast to other dipterans, including the mosquitoes *Aedes aegypti*, *Anopheles gambiae* and *Anopheles stephensi*, and the housefly, *Musca domestica*, in which a dominant male determining factor (M-factor) is the primary determinant of male differentiation^10–13^. However, M-factors identified in these species have no obvious sequence conservation, nor is their mode of function necessarily conserved. For example, in *M. domestica* the paternally inherited male determiner (*Mdmd*) *CWC22* paralog in XY embryos prevents the zygotic activation of the positive *Mdtra* loop, resulting in the default male-specific splicing of *Mdtra* and *Mddsx* pre-mRNAs leading to male differentiation^10^. The M-factors *Nix*, *Yob/gYG2* and *Guy1* from *A. aegypti*, *An. gambiae*, and *An. stephensi*, respectively, exhibit early embryonic transcription and subsequent expression throughout development in their respective species, and are required to initiate male development but have no primary sequence homology^11–13^. Since a *tra* ortholog sequence appears not to be conserved, and is possibly lacking in mosquito genomes, the different M-factors could more directly effect male-specific splicing of *dsx* pre-mRNA.

Y-linked M-factors have been inferred for several dipteran insects, but have not yet been identified in tephritid fruit flies, including the Oriental fruit fly, *Bactrocera dorsalis* (Hendel), which is one of the most devastating and highly invasive agricultural pests of fruits and vegetables throughout the world^14^. In contrast to *Drosophila*, the *tra* gene in *B*. *dorsalis* as well as other dipteran, hymenopteran and coleopteran species^10,15–20^, is able to autoregulate its female-specific splicing, analogous to the *Drosophila Sxl* gene. As maternal *tra* transcripts initiate a positive autoregulatory feedback loop in the early zygote, continuous female-specific functional TRA protein is provided and, thus, acts as a cellular memory, maintaining the female-determining signal^15^. For male development, the presence of an M-factor, which is required to prevent activation of the *tra* autoregulatory loop, leads to a male-specific nonfunctional TRA protein.

For *B*. *dorsalis*, and other tephritid pests, the foremost biologically-based population control strategy is the sterile insect technique (SIT), which relies on the ability to generate sterile male-only populations for mass release into infested areas^21^. Understanding the genetic basis of sex determination and male fertility has therefore been central to the development of genetically modified strains for sex separation and sterility. Although *Sxl* is not involved in tephritid sex determination, orthologs of the *Drosophila tra*, *transformer-2* (*tra-2*), and *dsx* genes are functionally homologous^5,7^. Therefore, the discovery of M-factors, or other upstream factors, remains fundamentally important for tephritid species. These factors may also include small RNAs as shown in the silkworm moth, *Bombyx mori*. In this lepidopteran species, a W chromosome-derived female-specific piRNA represses transcription of *Masculinizer* (*Masc*), leading to female-specific splicing of *Bmdsx* pre-mRNA in ZW females^22^. Thus, this piRNA acts as an essential primary feminizing factor in the ZW sex determination system. In view of this, we propose the hypothesis that small RNAs may also participate in sex determination by regulating the *tra* autoregulatory loop during early embryogenesis in *B*. *dorsalis*.

In this study, we identify the key embryonic stages in which male sex determination is initiated in *B. dorsalis*, and show that a small autosomally derived RNA, miRNA-1-3p, induces male sex determination by suppressing *Bdtra* expression during this developmental stage. Embryonic injection of a synthetic miR-1-3p inhibitor, or disruption of miR-1-3p by CRISPR/Cas9, induces a sex reversal of XY chromosomal males into phenotypic females that exhibit female-specific splicing of both *Bdtra* and *Bddsx* pre-mRNAs. Conversely, embryonic injection of a synthetic miR-1-3p mimic results in masculinization of XX individuals. These data support the conclusion that the miR-1-3p microRNA has a necessary intermediary function required for the induction of male sex determination in *B. dorsalis*.

## Results

### Analysis of small RNA libraries from embryonic stages

**Fig. 1:**
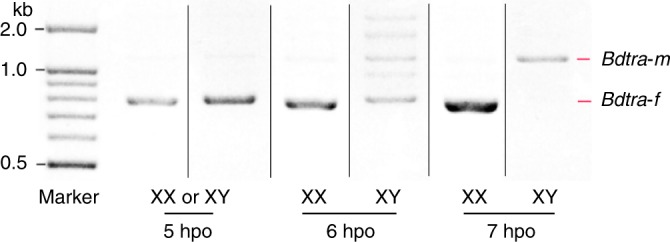
The splicing patterns of the *Bdtra* pre-mRNA transcript. Shown in this figure are RT-PCR products from single *B. dorsalis* embryos at 5, 6, and 7 h post-oviposition (hpo). *Bdtra-f* and *Bdtra-m* are the female and male-specific isoforms of the *transformer* transcript, respectively. The sex of each embryo is distinguished by the presence of *Bdtram* that is only expected in XY embryos. The samples derive from the same experiment and that gels were processed in parallel.

To identify factors that are involved in *B. dorsalis* sex determination, we first determined when *Bdtra-f* and *Bdtra-m* isoforms appear during embryogenesis. This was achieved by analyzing *Bdtra* sex-specific splicing isoforms in individual embryos at distinct hours post-oviposition (hpo) time points, thereby distinguishing the chromosomal sex of the embryos and their sexual fate. At 5 hpo, only female-specific *Bdtra* transcript was apparent in individual embryos, which was most likely due to maternal deposition of *Bdtra-f* in both XX and XY embryos. Male-specific *Bdtra-m* transcript was first detectable at 6 hpo while *Bdtra-f* transcript was not detectable at 7 hpo, presumably in XY male embryos. Embryos that expressed only *Bdtra-f* transcripts at 6 and 7 hpo were presumably XX (Fig. 1). These results suggest that the key stage for specifying male sexual development is between 5 and 6 hpo at 28 °C.

To identify candidate small RNAs that may be involved in sex determination, small RNA sequencing was performed using embryos of mixed sex at 5, 6, and 7 hpo (NCBI accession numbers: GSE117310). The three libraries produced a total of 6,178,809, 5,695,996, and 9,255,550 clean reads, respectively (Supplementary Table 1), and a total of 147 known and 103 novel miRNAs were identified in the combined data set (Supplementary Data 1 and 2). The majority of the small RNA reads ranged in size from 20 nt to 23 nt and had a peak at 22 nt, which is the typical length of mature miRNAs (Supplementary Fig. 1A). The first nucleotide position was enriched in uracil (U) with a frequency of 67% (Supplementary Fig. 1B), consistent with one of the most fundamental features of miRNA sequences^23,24^.

**Fig. 2:**
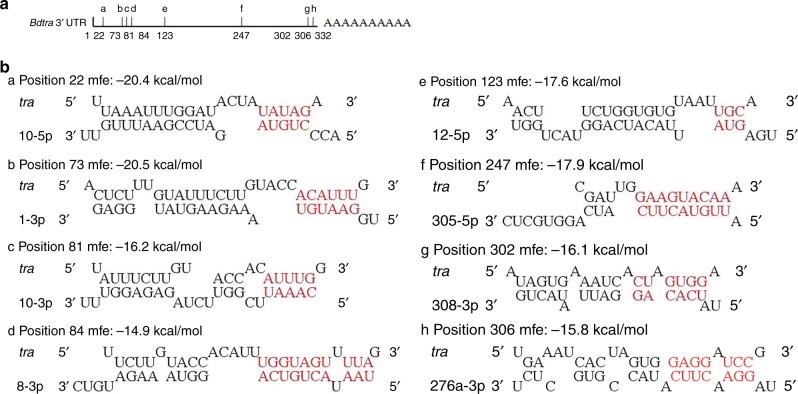
miRNAs that potentially target the *Bd*tra gene. **a** The position of miRNAs putative binding sites in the 3′ UTR of *Bdtra*, and (**b**) RNA-RNA duplexes of potential miRNA with the *Bdtra* gene as detected by RNA hybrids. miRNA seed sequences and their putative binding sites in the 3′ UTR are indicated by red. mfe: match free energy.

To study differential expression of known miRNAs within the three embryonic stages, the miRNA counts were normalized by transforming them into Reads Per Kilobase Per Million Mapped (RPKM). Sixty-five miRNAs were differentially expressed at 5, 6, and 7 hpo, according to a Fisher’s test and a chi-squared test (*P*-value < 0.05) (Supplementary Table 2). All 65 miRNAs, including miR-1-3p, are transcribed from autosomes and eight were found to potentially target the *Bdtra* gene (Supplementary Table 3). The putative target sequences of all eight miRNAs were found to be in the 3′ UTR of the *Bdtra* gene, with the smallest free energy value observed for miR-1-3p (Fig. 2; GenBank ID: JFBF01001330.1).

### miR-1-3p regulates *Bdtra* transcript accumulation

To test whether these eight miRNAs affect *Bdtra* expression in vitro, a dual luciferase assay was performed^25,26^. A firefly luciferase reporter construct was made by inserting the 3′ UTR of *Bdtra* downstream of the *Renilla* luciferase coding sequence of the psiCHECK-2 vector. To reduce interaction of endogenous miRNAs and mRNAs with our test system, we used a human cell line (HEK293T) for co-transfection with the dual-luciferase reporter and miRNA mimics. Whereas co-transfection with the miR-8-3p, miR-10-5p, miR-308-3p, miR-10-3p and miR-305-5p mimics did not significantly alter the luciferase signal, co-transfection with the miR-276a-3p, miR-1-3p and miR-12-5p mimics resulted in approximately 33%, 46% and 32% reductions in luminescence compared to controls, respectively (Supplementary Fig. 2). This suggested that only these three of the eight tested miRNAs targeted *Bdtra* in human cell lines.

**Fig. 3:**
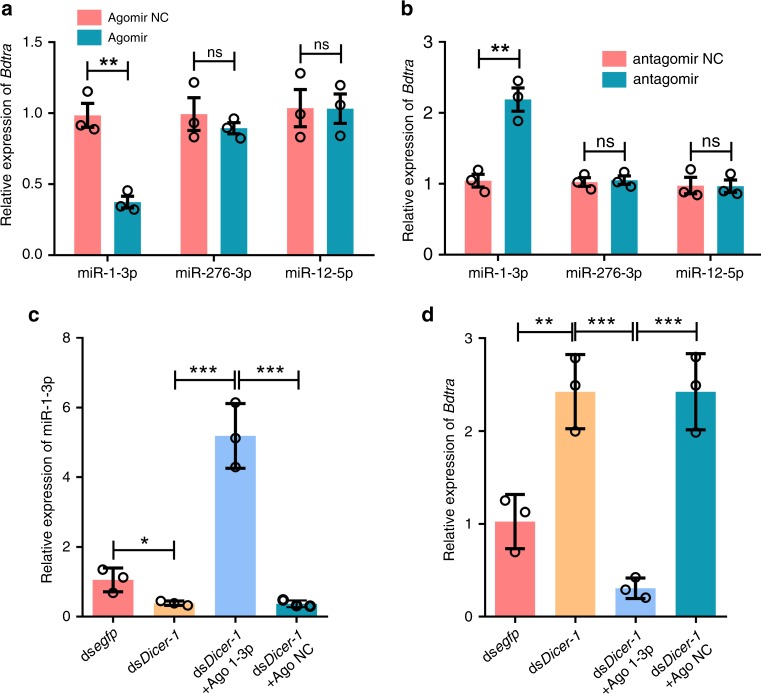
miR-1-3p targets the *Bdtra* transcript in vivo. **a** Levels of *Bdtra* in 10 h embryos injected with the miR-1-3p, miR-12-5p or miR-276a-3p agomir, (**b**) levels of *Bdtra* in 10 h embryos injected with the miR-1-3p, miR-12-5p or miR-276a-3p antagomir, (**c**) levels of miR-1-3p in 10 h embryos injected with ds*BdDicer-1* and the miR-1-3p agomir, and (**d**) levels of *Bdtra* in 10 h embryos injected with ds*BdDicer-1* and the miR-1-3p agomir. Error bars indicate the SEM of three independent biological replicates and asterisks (* and ** and ***) indicate the statistically significant differences (*P* < 0.05 and *P* < 0.01 and *P* < 0.001) between the treatment group and control group based on Student’s t-test. Ns indicates no statistically significant difference between the treatment group and the control group.

To determine the interaction between miR-276a-3p, miR-1-3p, miR-12-5p and *Bdtra* in vivo, we injected newly oviposited embryos (0–1 h) with corresponding miRNA agomirs (mimics), antagomirs (inhibitors) (Supplementary Fig. 3) and a *Bdtra* dsRNA/antagomir. Following injection of the miR-1-3p agomir, *Bdtra* mRNA decreased significantly by 62%, similar to the 68% decrease resulting from ds*Bdtra* injection in 10 h post-injection embryos (Fig. 3a and Supplementary Fig. 4A), whereas injection of the miR-1-3p antagomir inhibitor resulted in a 109% increase in *Bdtra* mRNA (Fig. 3b). Co-injection of antagomir-1-3p/ds*tra* decreased *Bdtra* transcript by 72%, whereas the control co-injection of antagomir-1-3p/ds*egfp* showed a similar increase in *Bdtra* transcript compared to the antagomir-1-3p treatments in 10 h embryos (Supplementary Fig. 4B). However, the expression level of *Bdtra* was not affected by miR-276a-3p and miR-12-5p agomir and antagomir treatments (Fig. 3a, b). Together, these results indicate that miR-1-3p down-regulates the level of *Bdtra* mRNA.

### miR-1-3p regulation of *Bdtra* is *BdDicer-1*-dependent

We analyzed the influence of depletion of *BdDicer-1*, the core component of the miRNA biogenesis pathway^27^, on miRNAs and *Bdtra* expression during embryogenesis. First, we analyzed the structure of *BdDicer-1* and assayed its expression. Structural analysis revealed that *BdDicer-1* contains an amino-terminal helicase domain, a PAZ domain, two RNAse III domains, and a dsRNA-binding domain (Supplementary Fig. 5). An expression profile of *BdDicer-1* transcript from different developmental stages showed that *BdDicer-1* is highly expressed in newly laid embryos of mixed sexes and showed increased levels at 6 and 7 hpo in early embryogenesis (Supplementary Fig. 6A, B).

To test the effect of *BdDicer-1* depletion on mature miRNA formation and *Bdtra* expression, we prepared a 643 bp dsRNA from the RNase III domains of *BdDicer-1*. The *BdDicer-1* RNAi results confirmed that transcriptional levels of the eight differentially expressed miRNAs were lower relative to ds*egfp* controls in 10 h embryos of mixed sexes. Among them, RNA levels of miR-1-3p, miR-8-3p and miR-308-3p significantly decreased by 56%, 58% and 51%, respectively (Supplementary Fig. 6C), whereas *Bdtra* mRNA levels in ds*BdDicer-1* treated embryos increased significantly by 137% compared to the ds*egfp* controls (Fig. 3d). A rescue experiment using miR-1-3p agomir in *BdDicer-1* knockdown individuals showed that miR-1-3p transcript increased greater than 12-fold in ds*BdDicer-1/*agomir-1-3p treated embryos of mixed sexes (Fig. 3c). In those embryos treated with both ds*BdDicer-1* and agomir-1-3p, the levels of *Bdtra* decreased significantly by 87% at 10 h post-injection, whereas co-injection of ds*BdDicer-1*/agomir negative control (NC) had a similar effect on *Bdtra* expression compared to the ds*BdDicer-1* treatment alone (Fig. 3d). These results demonstrate that *BdDicer-1* is required for miR-1-3p biogenesis and, hence, for the reduction of *Bdtra* mRNA levels induced by miR-1-3p.

### miR-1-3p is required for male sex determination in embryos

**Fig. 4:**
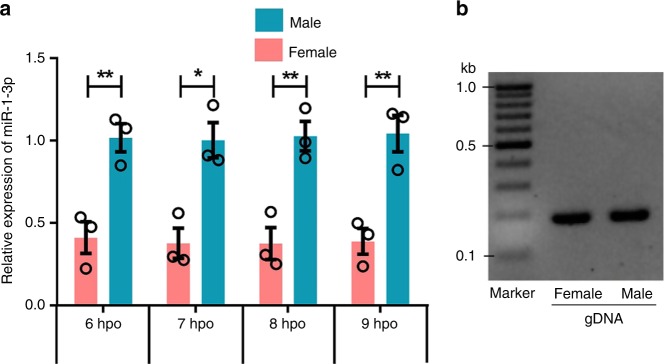
The differential expression of miR-1-3p in female and male embryos. **a** Relative levels of miR-1-3p in single female and male embryos at 6, 7, 8, and 9 h post-oviposition (hpo). Female and male embryos were identified by the splicing patterns of *Bdtra* pre-mRNA. (**b**) PCR amplification of miR-1-3p from adult female and male genomic DNA. Error bars indicate the SEM of three independent biological replicates and asterisks (**) indicate the statistically significant differences (*P* < 0.01) between the treatment group and control group based on Student’s *t* test.

Based on the detection of only *Bdtra-m* in single XY embryos, which is the major characteristic of sex-specific splicing of *Bdtra* pre-mRNA, we identified the sex of individual female and male embryos at 6, 7, 8, and 9 hpo. The expression pattern of the miR-1-3p gene in 0–9 hpo embryonic stages, including mixed embryos at 0, 1, 2, 3, 4, 5 hpo, and single female and male embryos at 6, 7, 8, 9 hpo, showed that miR-1-3p levels increased at 0 hpo to 6 hpo. Levels increased more so from 5 hpo to 6 hpo in mixed embryos (Supplemental Fig. 7A), and the relative expression levels of miR-1-3p in single male embryos were significantly higher than in single female embryos (Fig. 4a). The temporal profile in immature (IM), middle-aged (MA) and fully mature (FM) female and male adults confirmed that miR-1-3p has sexually dimorphic expression also during adulthood (Supplemental Fig. 7B). Our sequencing data indicated that miR-1-3p, having the Genbank ID: JFBF01001330.1, is transcribed from an autosome and is not X-linked (Supplementary Data 1). Furthermore, miR-1-3p PCR amplification from adult female and male genomic DNA also confirmed that miR-1-3p is not sex-specific and, thus, not linked to the male Y chromosome (Fig. 4b). These results indicated that a sexually dimorphic switch in miR-1-3p expression results in male-specific splicing of *tra* between 5 and 6 hpo and, thus, has a role in inducing male sex determination in the XY embryo.

**Fig. 5:**
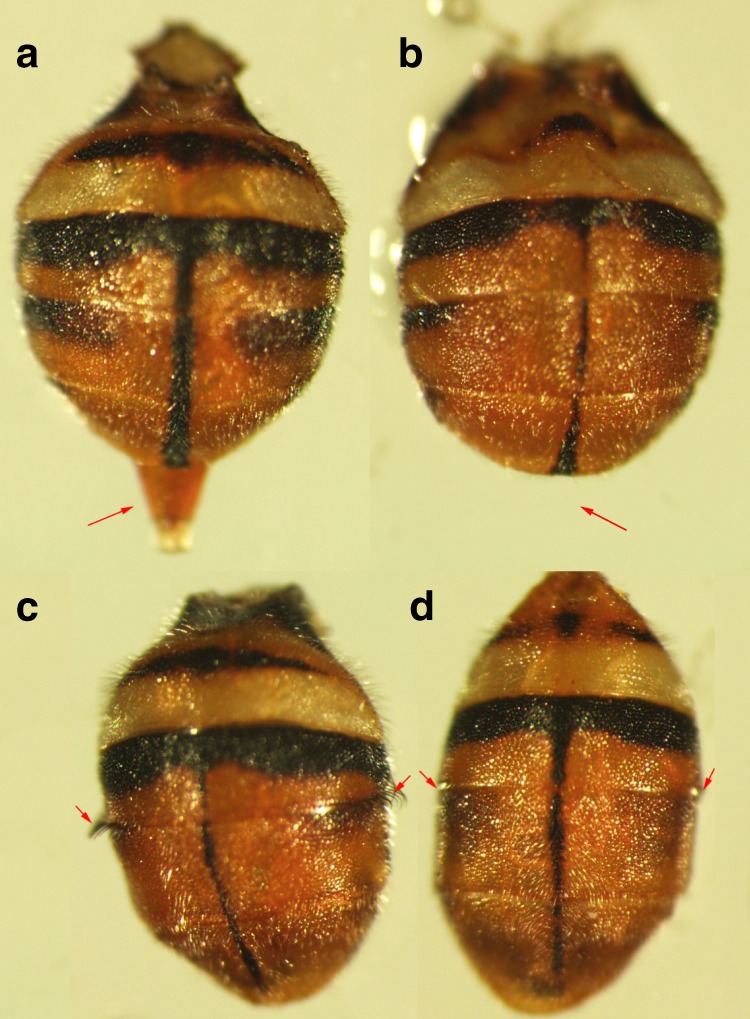
Dorsal view of abdominal intersex phenotypes induced by embryonic injection. **a** Wild type female adult, (**b**) intersex of incomplete masculinization individuals exhibiting a female dorsal phenotype but lacking the ovipositor after miR-1-3p agomir injection, (**c**) dorsal overview of WT male adult, and (**d**) intersex due to incomplete feminization exhibiting a male dorsal phenotype without bristles on the side of the third tergum after the miR-1-3p antagomir and sgRNA injection.

**Fig. 6:**
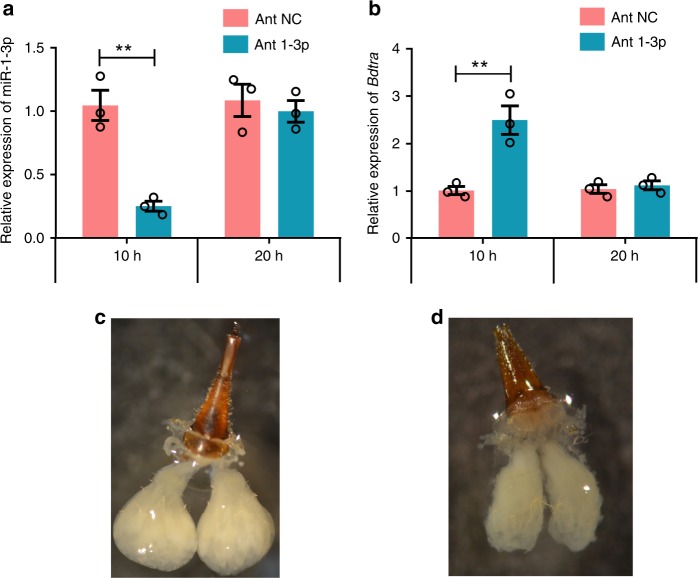
Embryonic silencing of miR-1-3p leads to incomplete feminization. **a** Relative expression levels of miR-1-3p transcript in embryos 10 h and 20 h after injection of the miR-1-3p antagomir, (**b**) relative expression levels of *Bdtra* transcript in embryos 10 h and 20 h after injection of the miR-1-3p antagomir, (**c**) dissected normal ovaries from a female injected with the antagomir negtive control (NC), and (**d**) dissected partially feminized ovaries from a female injected with the miR-1-3p antagomir. Error bars indicate the SEM of three independent biological replicates and asterisks (**) indicate the statistically significant differences (*P* < 0.01) between the treatment group and control group based on Student’s *t* test.

To investigate the role of miR-1-3p in determining male sex in early embryos, wild type embryos were injected with agomir-1-3p and antagomir-1-3p. We found that pooled embryos injected with agomir-1-3p resulted in progeny composed of 87–92% phenotypic males and pools injected with antagomir-1-3p resulted in 67–77% phenotypic females, whereas the female to male ratio of uninjected, agomir NC/antagomir NC control, and miR-1-3p mutant agomir/antagomir injected eggs was approximately 1:1 (Supplementary Table 4, 5). Two to 4% intersexual individuals exhibited a female dorsal phenotype, lacking an ovipositor, in agomir-1-3p injected pools and 2–6% intersexual individuals exhibited a male dorsal phenotype by the absence of bristles on the side of the third tergum in antagomir-1-3p injected pools (Fig. 5 and Supplementary Table 4). The low feminization rate of antagomir-injected embryos may be explained by the transient nature of embryonic artificial miRNA interference. A 76% reduction of miR-1-3p transcript levels was observed in embryos 10 h after antagomir-1-3p injection, whereas after 20 h, levels were comparable to those in control individuals, suggesting a recovery of miR-1-3p expression (Fig. 6a). The expression of *Bdtra* increased 147% in embryos 10 h after antagomir-1-3p injection with no significant difference at 20 h after injection (Fig. 6b). The incompletely feminized intersexual individuals had immature ovaries with non-vitellogenic oocytes, whereas the normal females had normal vitellogenic ovaries with mature oocytes (Figs. 6c, d).

**Fig. 7:**
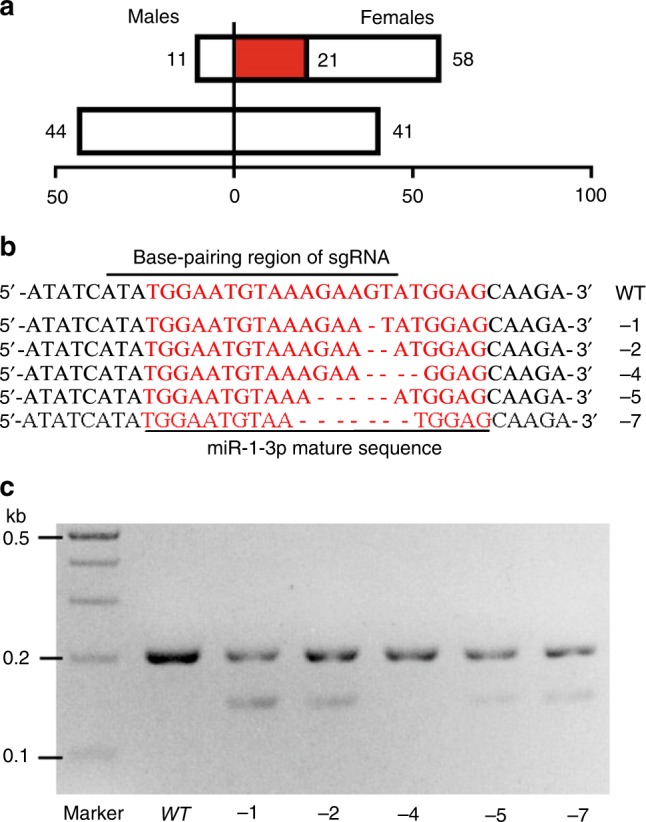
CRISPR/Cas9 induced genomic disruption of miR-1-3p. **a** The sexual phenotype of wild type (*wt*) adults having an miR-1-3p knock-out deletion (miR-1-3p^−^) mediated by CRISPR/Cas9. CK is the control group injected with Cas9-mRNA and *egfp* sgRNA. Individuals with mutations in miR-1-3p target sites (red bar) are presumed to be feminized XY chromosomal males based on an expected 1:1 sex ratio and the number of estimated true XX chromosomal females (white bar). (**b**) Deletion products in five G0 flies (−1, −2, −4, −5, and −7 deletion mutations) resulting from non-homologous end-joining of the targeted genomic sequence by miR-1-3p sgRNA. (**c**) PCR products amplified from WT and mutant G0s treated with T7 Endonuclease I (T7EI) where additional shorter products detected in mutants represent the different miR-1-3p deletions.

To further test whether miR-1-3p is required for male sex determination, miRNA-specific mutations were generated by CRISPR/Cas9 gene-editing (Supplementary Fig. 8). The 706 embryos injected together with Cas9-mRNA and miR-1-3p sgRNA resulted in 72 surviving adults comprised of 58 females, 11 males and 3 intersexual individuals, yielding a female to male sex ratio of 6:1. In contrast, 722 embryos injected with Cas9-mRNA/*egfp* sgRNA resulted in 85 adult survivors having a phenotypic female to male ratio of nearly 1:1 (41 females: 44 males) (Fig. 7a and Supplementary Table 6). A PCR-based analysis using DNA extracted from G0 individual adults, injected with Cas9/sgRNA as embryos, showed that 21 of the 58 G0 females and 3 intersexual individuals had indels in the targeted miR-1-3p region, whereas none of the 11 male adults exhibited miR-1-3p mutations, nor did the control injected individuals. Deletions ranged from 1 bp to 7 bp in the miR-1-3p mutated sequence (Fig. 7b) and a T7 Endonuclease I (T7EI) assay confirmed that PCR fragments spanning the miR-1-3p target sites could be cut into expected size bands in mutant individuals (Fig. 7c). This is consistent with a genomic disruption of miR-1-3p by the CRISPR/Cas9 system resulting in some chromosomal XY male individuals undergoing a sex reversal into phenotypic females.

**Fig. 8:**
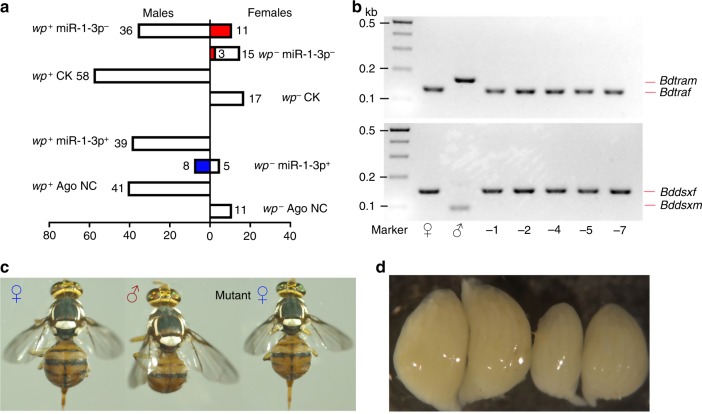
miR-1-3p is sufficient to initiate male development in *B. dorsalis*. **a** The sexual phenotype of the *white/brown pupae* sexing strain (*wp*^*+/−*^) adults having an miR-1-3p knock-out deletion (miR-1-3p^−^) mediated by CRISPR/Cas9 and after embryonic injection of the miR-1-3p mimic (miR-1-3p^*+*^). *wp*^*+*^ are genetic males (XY) and *wp*^*−*^ are genetic females (XX). The red bar represents the number of miR-1-3p mutant adults, and the blue bar represents the number of phenotypic male adults which developed from the *white pupae* (XX *wp*^*−*^) embryos treated with miR-1-3p agomir. NC is the control group injected with the agomir negative control. (**b**) Sex-specific transcripts of *Bdtra* and *Bddsx* in WT females and males and XY miR-1-3p mutant phenotypic females. −1, −2, −4, −5, and −7 represent different types of miR-1-3p mutant females. (**c**) Phenotypes of XX *wp*^*−*^ female (left) and XY *wp*^*+*^ male (middle) adults from the *white/brown pupae* sexing strain, and an XY *wp*^*+*^ miR-1-3p mutant phenotypic female (right) induced by CRISPR/Cas9. (**d**) Mature vitellogenic ovaries from an XX *wp*^*−*^ female (left) and an XY *wp*^*+*^ miR-1-3p mutant phenotypic female (right).

**Fig. 9:**
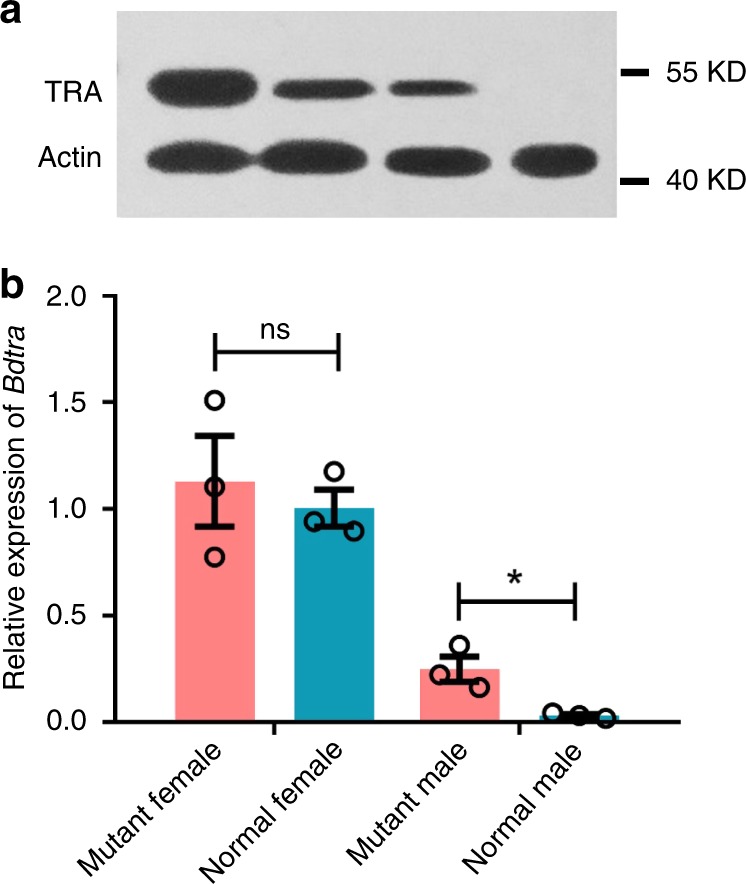
The effect of miR-1-3p on *Bdtra* expression. **a** A Western blot showing BdTRA protein levels in normal and miR-1-3p mutant females and males, and (**b**) relative *Bdtra* mRNA levels by qPCR in normal and miR-1-3p mutant females and males. Error bars indicate the SEM of three independent biological replicates and asterisks (*) indicate the statistically significant differences (*P* < 0.05) between the treatment group and control group based on Student’s *t* test. Ns indicate no statistically significant difference between treatment group and the control group.

However, we could not definitively determine the chromosomal sex of the 21 mutant phenotypic females (Fig. 7a), which was achieved by using the *white/brown pupae* strain developed for *B. dorsalis* sexing in the sterile insect technique^28^. In this strain, XX individuals homozygous for the recessive *white pupae* (*wp*^−^) mutation develop as non-pigmented white pupae, whereas XY individuals, having a wild type *wp* allele (*wp*^+^) translocated to the Y chromosome, develop as normally pigmented brown pupae (Supplementary Fig. 9). Thus, the CRISPR/Cas9 experiment was repeated in this strain to confirm the feminization of brown pupal XY males as a result of mutations in miR-1-3p (Fig. 8a, c). In the experimental group, 714 embryos injected with miR-1-3p sgRNA/Cas9 resulted in 62 surviving adults consisting of 26 phenotypic females and 36 phenotypic males (Fig. 8a and Supplementary Table 7). Of these adults, 47 developed from XY brown pupae, 11 of which were sexually reversed phenotypic XY females (Fig. 8a) that carried indel mutations in the targeted miR-1-3p region (Figs. 7b, c). They also expressed the female splice variants of *Bdtra* and *Bddsx* and had normal ovaries (Fig. 8b, d). The remaining 36 adults developing from XY brown pupae were normal phenotypic males. Among 15 adult females that developed from XX white pupae, three had miR-1-3p mutations and expressed the normal female splice variants of *Bdtra* and *Bddsx*. The backcross experiment of the 11 feminized XY females mated to normal XY males showed that eight (73%) mated normally, both the number of eggs per female and their hatching rate were similar to control injected (*egfp* sgRNA/Cas9) females (Supplementary Fig. 10), and the F1 adult sex ratios had no significant bias to either sex (Supplementary Table 8). The 729 control embryos injected with *egfp* sgRNA/Cas9 resulted in 75 non-mutant adult survivors (Fig. 7a and Supplementary Table 6) consisting of 17 phenotypic females and 58 phenotypic males, having an approximate 1:3 sex ratio (as observed in the non-treated strain, consistent with Mendelian segregation of the dominant marker). The mRNA and protein levels of *Bdtra* were also investigated in normal and miR-1-3p mutant female and male individuals by qPCR and Western blots. The results showed that both mRNA and protein levels of *Bdtra* gene were female-specific in mutant feminized XY males, whereas they were hardly detectable in normal males (Figs. 9a, b). Taken together, these results show that CRISPR/Cas9 embryonic mutagenesis of miR-1-3p in XY individuals confers a complete male to female sex reversal consistent with its requirement for male development.

To further confirm that the miR-1-3p is required for masculinization, we investigated the influence of miR-1-3p overexpression on sexual differentiation by injection of agomir-1-3p into embryos of the *white/brown pupae* sexing strain. We observed 52 surviving adults consisting of 39 XY (*wp*^*+*^) brown phenotypic males, 5 phenotypic XX (*wp*^*−*^) white females, and 8 XX (*wp*^*−*^) white phenotypic males (Fig. 8a and Supplementary Table 9). The backcross experiments of the 8 masculinized XX males mated to the normal XX females, showed that five (63%) mated normally, and both the number of eggs per female and their hatching rate were similar to the control injected (agomir NC) treatment (Supplementary Fig. 11) and the majority of F1 progeny were female (Supplementary Table 10). For a control test, 697 *white/brown pupae* sexing strain embryos were injected with agomir NC, which resulted in 52 adult survivors consisting of 11 phenotypic females and 41 phenotypic males, with all males developing from brown pupae (Fig. 7a and Supplementary Table 9), consistent with Mendelian segregation of the dominant marker. Thus, the masculinization of XX white pupae by agomir-1-3p treatment indicates that the induced overexpression of miR-1-3p is sufficient to initiate male development in *B. dorsalis* in the absence of the Y chromosome.

## Discussion

The primary molecular signals that determine sex are highly divergent in insects, but in several Orders they act on a functionally conserved key female-determining gene, *transformer*^29–31^. In this study, we showed that 5 to 6 hpo is a key stage for initiating male sex determination in *B. dorsalis*, during which the male-specific *Bdtra-m* transcripts appeared and the female-specific *Bdtra-f* transcripts degraded and became undetectable by 7 hpo at 28 °C. This timing is similar to that found in the other *Bactrocera* species, *B. jarvisi* and *B. tryoni*, where degradation of maternally deposited *tra-f* begins at 5–6 hpo and 9 hpo in male embryos at 25 °C, respectively^32^, whereas in *C. capitata* it occurs at 6-7 hpo at 25 °C ^33^. This time period for establishing the sex-determined state is coincident with the stage at which the proposed M-factor is likely to be active, for example, in maternal *tra* degradation^32,33^. The establishment of male sex determination during early embryogenesis in *Bactrocera* species, is also coincident with a similar sex-determining developmental stage in other insect species, including the dipterans, *D. melanogaster*^34^ and *M. domestica*^35^, the lepidopteran *B. mori*^22^, the coleopteran *Tribolium castaneum*^36^, and the hymenopterans, *Apis mellifera*^37^ and *Nasonia vitripennis*^38^.

microRNAs (miRNAs) are small noncoding RNAs that regulate spatio-temporal gene expression in various biological processes, including the degradation of maternal mRNAs in the developing embryo^39^. In our study, both the in vitro degradation of *Bdtra* 3′ UTR-contained luciferase reporter by a miR-1-3p mimic and the alteration of *Bdtra* transcript level by a synthetic miR-1-3p mimic and inhibitor in vivo confirmed that miR-1-3p could target *Bdtra* in early embryogenesis. Our RNAi tests indicated that silencing of *BdDicer-1* in *B. dorsalis* embryos resulted in reduced levels of mature miRNAs, including miR-1-3p, and increased levels of *Bdtra* mRNA, indicating a link between *BdDicer-1*, miR-1-3p and regulation of *Bdtra* transcript level. Reduction in the level of *Bdtra* mRNA was also observed in *BdDicer-1*-RNAi experiments when additionally treated with a miR-1-3p mimic, suggesting that its action on *Bdtra* is independent of RNAi.

In order to verify this hypothesis for the requirement of miR-1-3p for male sex determination, miR-1-3p-specific mutations were generated in a wild type strain and *white/brown pupae* sexing strain having a Y-linked marker in *B. dorsalis*. The latter *wp* strain, in particular, showed that the miR-1-3p specific mutation in XY individuals results in female expression of *Bdtra* and complete adult feminization, whereas XX females developed normally. This proposed role for miR-1-3p in *B. dorsalis* male sex determination would be the converse of the proposed role for the *Fem* piRNA in *B. mori*, as the primary female-determining factor in the ZW/ZZ sex determination system. Here, the *Fem* piRNA represses *Masc* mRNA expression required for male *Bmdsx* pre-mRNA splicing and male sexual development^22^. Nevertheless, the roles of miR-1-3p miRNA and *Fem* piRNA in the regulation of *tra* and *Masc*, respectively, indicate that small RNAs, in different orders of insects, have essential roles as post-transcriptional factors during the initiation of sex determination in early embryogenesis. However, miR-1-3p is a non-Y chromosome-linked miRNA with higher transcript levels in early XY embryos, which differs from *Fem* piRNA which is expressed from the female-specific W chromosome in *B. mori*^22^. The dominant component of the silkworm W chromosome is female-enriched piRNAs gene clusters^40^, whereas the major component of tephritid Y chromosomes is repeat-rich sequences, as reported for *C. capitata*^41,42^. Indeed, the diversity of heterogametic sexual determinants between *B. mori* and *B. dorsalis* may explain, in part, the differing roles for small RNAs and mRNAs in regulating sex determination in the two species. Our study extends this pathway of interactions, at least for a tephritid species, by showing that miR-1-3p is a transducer of the Y-linked primary signal for male sex determination and may play a more direct role as a male-determiner by promoting male-specific *tra* pre-mRNA splicing and, hence, its functional inactivation in XY embryos.

**Fig. 10:**
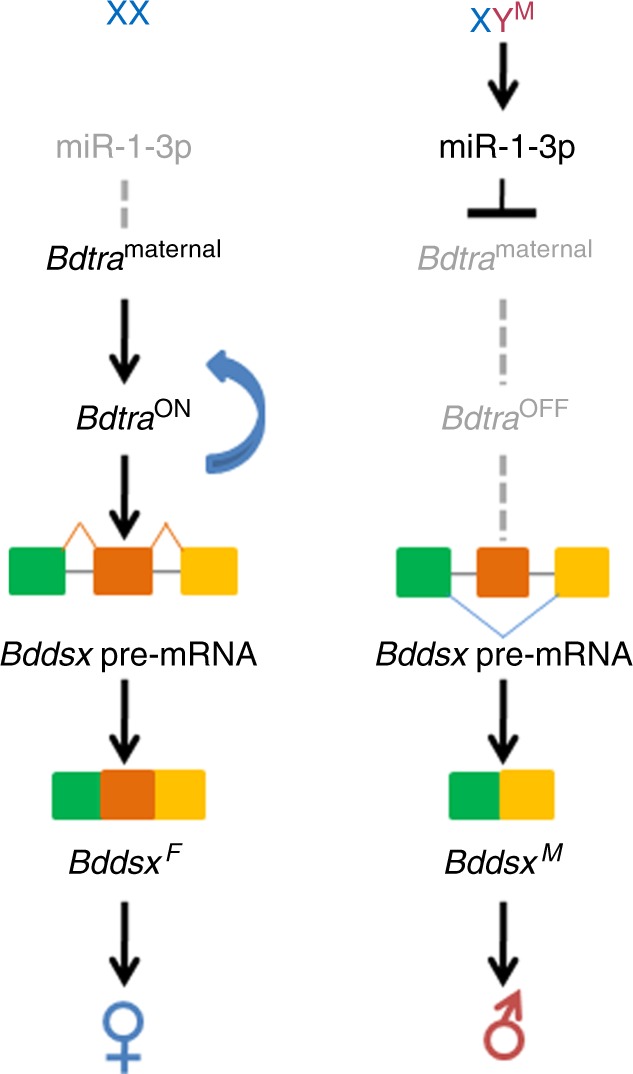
A proposed model for the sex determination pathway in *B. dorsalis*. In XY embryos, miR-1-3p is activated by the Y-linked primary signal (M) to inhibit maternal *Bdtra* resulting in male-specific splicing of *Bdtra* pre-mRNA. Thus, a lack of BdTRA leads to *Bddsx-m* splicing resulting in BdDSXM expression that directly promotes male sexual differentiation. In XX embryos low expression levels of miR-1-3p results in insufficient inhibition of maternal *Bdtra* transcript, allowing it to translate into sufficient functional BdTRA protein to initiate the female sexual fate autoregulatory loop in zygotes. BdTRA protein then activates the female-specific splicing of the downstream *Bddsx*^*F*^ gene pre-mRNA, thus, promoting female sexual differentiation. The components that are inactive are shown in gray.

These results lead us to propose an updated model for the sex determination pathway in *B. dorsalis* (Fig. 10), that might be extended to related tephritid species^15–19,43^. Since the miR-1-3p gene is present in both sexes, albeit only transcribed at high levels in male embryos during a developmental period critical to sex determination, and is thus not Y-encoded, this model proposes that its expression is activated in early embryogenesis by a Y-linked primary signal in male embryos. This primary signal, is the putative male determining factor (M-factor), whereas miR-1-3p has a more direct role in inhibiting maternal *Bdtra* expression. The inhibition of maternal *Bdtra* expression would result in male-specific splicing of zygotic *Bdtra* pre-mRNA, producing a truncated BdTRA unable to maintain the threshold level needed to sustain the feedback loop of *Bdtra* expression. Thus, a lack of BdTRA protein in XY would lead to *Bddsx-m* splicing and the production of the BdDSXM protein that programs male sexual differentiation. However, due to low expression levels of miR-1-3p in female embryos, insufficient inhibition of maternal *Bdtra* transcript would allow it to translate into functional BdTRA protein to initiate the female-specific autoregulatory loop in the zygotes. BdTRA, in concert with BdTRA-2, would then result in female-specific splicing of the downstream *Bddsx* gene pre-mRNA, thus promoting female sexual differentiation.

In summary, we have shown that miR-1-3p is essential in determining the male sex in *B. dorsalis* through a combination of miR-1-3p knockdown, knockout and over expression experiments. This is the first report of a direct role for a miRNA-mediated sex determination mechanism in insects, and the second finding of a role for a small RNA, although the *B. mori* piRNA and miR-1-3p act conversely. This suggests that small RNAs may have an expanded and varying repertoire of regulatory functions for sex specification in insect species, and perhaps other organisms. For *B. dorsalis* in particular, manipulation of miR-1-3p may be used for sexing in SIT, for example, by producing male-only populations by its conditional ectopic overexpression in XX females during embryogenesis.

## Methods

### Insect rearing

*B. dorsalis* wild type and *white/brown pupae* sexing (provided by Dr. Eric Jang, USDA-ARS, Hilo, HI) strains were reared at the Institute of Horticultural and Urban Entomology at Huazhong Agricultural University (Wuhan, China). Adult flies were fed an artificial diet consisting of yeast extract and sugar and cultured at 28 °C under a 12 h light: 12 h dark photoperiod in cages, and hatched larvae were maintained on bananas^18^. The sex ratio of normally bred adult females to males is approximately 1:1 for wild type and 1:3 for the *white pupae* strain. We have complied with all relevant ethical regulations for *B. dorsalis* testing and research.

### Sample preparation

Embryo total RNAs were extracted using RNAiso Plus reagent (TaKaRa, Japan) according to the manufacturer’s protocol. First strand cDNA was synthesized from 1ug total RNA using PrimeScript^®^ Reverse Transcriptase (TaKaRa, Dalian, China) with the oligo (dT) adapter primer. Female and male transcripts from *Bdtra* were amplified by RT-PCR from 5, 6, 7, 8, and 9 h post-oviposition (hpo) embryo cDNA with *Bdtra* sex-specific exon primers (Supplementary Table 11). Embryos at 5, 6 and 7 hpo were collected and stored in RNAlater^®^ Solution (Ambion, Austin, TX, USA). To identify the small RNAs involved in *B. dorsalis* primary sex determination, three small RNA libraries were constructed from the 5, 6 and 7 hpo samples. Total RNAs were extracted using RNAiso Plus reagent (TaKaRa, Dalian, China) according to the manufacturer’s protocol, with the quantity and purity examined using a Bioanalyzer 2100 (Agilent, CA, USA).

### Small RNA library construction and deep sequencing

Small RNA libraries were generated from the three RNA samples using the TruSeq Small RNA Sample Prep Kits, following manufacturer’s guidelines (Illumina, San Diego, USA). The 18-30 nt length RNA fractions were separated by gel electrophoresis and ligated sequentially to 3′ and 5′ adapters. Ligation products were then reverse transcribed using a primer to the 3′ adapter with 15 PCRs amplified for the first strand synthesis production with both adapter sequence primers. Ultimately, PCR products were purified, validated and sequenced using an Illumina HiSeq 2500 System (LC-Sciences, Hangzhou, China).

### Small RNA sequence analysis

After removing low quality reads and adapter sequences from the raw data, large amounts of clean small RNAs were analyzed by BLAST against the *B. dorsalis* mRNA database^44^, RFAM and Repbase to identify possible mRNA, rRNA, tRNA, snRNA, snoRNA and other ncRNAs. The remaining clean reads were aligned to the miRNA precursors/mature miRNA sequences in miRBase and previously identified miRNAs of *B. dorsalis*^45^, with the matched or single mismatch sequences, were identified as known miRNAs. Unmapped sequences were aligned to the whole genome sequence (WGS) of *B. dorsalis* (NCBI Assembly# ASM78921v2) and hairpin RNA structures containing sequences were predicted from flanking 80 nt sequences using RNAfold software (http://rna.tbi.univie.ac.at/cgi-bin/RNAfold.cgi) complying with criteria from pre-miRNA statistics in miRBase to identify potentially novel miRNAs.

### Comparison of differentially expressed miRNAs

As male sex determination occurs within 5, 6, and 7 hpo, the expression patterns of miRNAs during these three key embryonic stages were compared to identify differentially expressed miRNAs which target the *Bdtra* gene. The abundance of each miRNA in the libraries was transformed through a modified global normalization. Normalization calculation was performed to adjust for the three sequencing libraries. The normalized data were used to calculate fold-change values [=log_2_(X/Y)] and the log_2_-ratio plot. The statistically significant difference between each library was tested with a Fisher’s test and a chi-squared test.

### Target gene prediction

For the prediction of miRNAs targeting the *tra* gene in *B. dorsalis*, three different target prediction programs were used: miRanda^46^, RNAhybrid^47^, and TargetScan^23^. To characterize the interaction of the *Bdtra* gene (GenBank ID: KP769559.1) with differentially expressed miRNA candidates, the full length sequence of the 3′ UTR of *Bdtra* and miRNA sequences were submitted on-line to RNAhybrid, an algorithm taking into account the free energy level of RNA-RNA duplexes and degree of miRNA target sequence complementarity using default parameters^47^.

### Cell culture and dual luciferase reporter (DLR) assay

HEK293T cells provided by Prof. Shengbo Cao (Laboratory of Animal Virology, College of Veterinary Medicine, Huazhong Agricultural University) were cultured and maintained in Dulbecco’s Modified Eagle’s Medium (Gibco, Carlsbad, CA, USA) supplemented with 10% fetal bovine serum (Gibco, Carlsbad, CA, USA), and 1% penicillin/streptomycin (Gibco, Carlsbad, CA, USA) at 37 °C in 5% CO_2_.

The *Bdtra* 3′ UTR (332 bp) was amplified by PCR from *B. dorsalis* cDNA and inserted into the psiCHECK-2 vector (Promega, Madison, WI, USA) which contains the firefly and renilla luciferase gene using *Not* I and *Xho* I sites, respectively (see sub-cloning PCR primers in *SI Appendix*, Table S8). After growth in 96-well plates with serum-containing medium for 12 h (10^4^ seeded cells per well), cells were transfected with 50 nM miRNA agomir or the negative control (NC) and 100 ng luciferase reporter vector psiCHECK-2, containing the *Bdtra* 3′ UTR, in each well using 0.3 μl FuGENE^®^ HD (Promega, Madison, WI, USA). 48 h after transfection, cells were lysed and the activity of firefly and renilla luciferase was determined with the Dual-Luciferase^®^ Assay System (Promega, Madison, WI, USA) according to the manufacturer’s protocol. Firefly luciferase values were normalized to renilla, and the ratio of firefly/renilla was presented.

### miRNA agomir/antagomir and dsRNA embryo microinjection

A 598 bp fragment of the *Bdtra* gene and a 643 bp fragment of the *BdDicer-1* gene (RNase III coding region) were produced by PCR, and a control *egfp* fragment was amplified with primers from the *egfp*-containing vector pB[PUbnlsEGFP]^48^ (Supplementary Table 11). Double stranded RNA (dsRNA) from *Bdtra, BdDicer-1* and *egfp* fragments were synthesized using the T7 RiboMAX™ Express RNAi System (Promega, Madison, WI, USA), purified in elution buffer by MEGAclear (Ambion, Austin, TX, USA) and stored at −20 °C until use. miRNA unique RNA-based mimics (agomirs), inhibitors (antagomirs) and negative control (NC) non-target oligonucleotides were synthesized by GenePharma (Shanghai, China). miRNA agomir and antagomir sequences are listed in Supplementary Table 12.

Newly oviposited embryos (0–1 hpo) were collected and microinjected with 100 uM miRNA agomir/antagomir and mutuant agomir/antagomir or 2 ug/ul ds*Dicer-1/*ds*tra*, or a mixture of 100 μM agomir/antagomir and 2 μg/ul dsRNA according to the transformation procedure described by Sharma et al.^10^. Equivalent volumes of NCs and ds*egfp* were injected for controls. Ten hours post-injection, ten embryos from each treatment group were assayed for expression of miR-1-3p and *Bdtra* that was replicated at least three times for each treatment group. In miR-1-3p agomir/antagomir injection experiments embryos were incubated in an oxygen-saturated container at 28 °C. Surviving larvae were collected and placed on banana media until eclosion. Adults were collected and their emergence rate, sex ratio, and morphological changes were recorded.

### In vitro synthesis of Cas9-mRNA and sgRNA

A miRNA-targeted genomic mutation was created by CRISPR/Cas9 using a miR-1-3p specific sgRNA according to the methods of Ling et al.^25^. and Zhang et al.^26^. To design the miR-1-3p specific sgRNA, miR-1-3p mature regions were searched with the protospacer-adjacent motifs (PAMs) of the recognition site for *Streptococcus pyogenes* Cas9 (NGG) (Supplementary Fig. 8). The base-pairing region was 20 bp that excludes the PAM sites to achieve high targeting accuracy. Potential off-target binding was assessed using the CasOT search tool and the direct BLAST of selected sequences against the *B. dorsalis* genome sequence (NCBI Assembly# ASM78921v2), which revealed no off-target sites suggesting a high specificity of the miR-1-3p sgRNA. The dsDNA template for sgRNA synthesis was generated from template-free PCR, where one specific forward primer and one universal reverse primer overlapped (primers listed in Supplementary Table 11). T7-Cas9 plasmids (V-solid, Beijing, China) were linearized by *Not* I and recovered as corresponding templates to obtain in vitro transcription of Cas9-mRNA. The dsDNA template and endonuclease digestion products were purified using the E.Z.N.A.^®^ Cycle-Pure Kit (Omega, Norcross, GA, USA). In vitro transcription of sgRNA and Cas9-mRNA were carried out following the instructions of the MEGAscript^®^ Kit (Ambion, Austin, TX, USA). The sgRNA and Cas9-mRNA were purified with the MEGAclear Kit (Ambion, Austin, TX, USA) and stored at -80 °C until use. Embryos were microinjected with a mixture of purified Cas9-coding mRNA and miR-1-3p sgRNA according to the microinjection protocol described above. Final concentrations of Cas9-coding mRNA and miR-1-3p sgRNA were 300 ng/ul and 150 ng/ul respectively and Cas9-coding mRNA and *egfp* sgRNA were used as controls. Surviving larvae were placed on banana media until adult eclosion at which time G0 adults were collected with their emergence rate, sex ratio and morphology changes recorded.

### Fertility tests and mutagenesis analysis

Phenotypic females that developed from chromosomal male (Y, *wp*^+^) brown pupae and phenotypic males that developed from chromosomal female (*wp*^−^) white pupae were reared and tested for fertility under standard laboratory conditions. Each female was backcrossed to three virgin normal XY males and each male was backcrossed to three virgin normal XX females in separate cages. Matings were observed at 30 min intervals during the dark photoperiod, and eggs from each cage were collected and counted continuously for 5 days. The newly hatched larvae were counted and then transferred onto banana until adult emergence. To molecularly characterize CRISPR/Cas9 induced mutations, genomic DNA was extracted from G0 individuals following the E.Z.N.A.^®^ Insect DNA Kit protocols (Omega, Norcross, GA, USA). The miR-1-3p target loci were amplified by PCR using the following conditions: 94 °C for 3 min, 35 cycles of 94 °C for 30 s, 55 °C for 30 s, and 72 °C for 30 s, followed by a final extension at 72 °C for 10 min. PCR products were cloned into *pEASY*^®^-T1 vector (TransGen Biotech, Beijing, China) and sequenced using GenScript (Nanjing, China). The PCR products were also used for the T7 endonuclease I (T7EI) assay according to the manufacturer’s instructions (V-solid, Beijing, China). For expression analysis of the *Bdtra* and *Bddsx* genes, total RNA was extracted and cDNA prepared from a G0 individual. Primers that were designed to detect mutagenesis in targeted sites and sex-specific expression of *Bdtra* and *Bddsx* are listed in Supplementary Table 11.

### Quantitative real-time PCR of mRNA and miRNA

Quantitative real-time PCR (qPCR) was used to assay the developmental transcript expression profiles of *BdDicer-1* in 0, 5, 6, and 7 hpo embryos, 1st, 2nd, and 3rd instar larvae, early and late stage pupae, and 1 d and 10 d female and male adults. qPCR was also used to test transcript expression levels of miR-1-3p and *Bdtra* in miR-1-3p agomir/antagomir and ds*tra*/ds*Dicer-1* treated individuals. Total RNA was extracted using RNAiso Plus reagent (TaKaRa, Dalian, China), with 200 ng for each sample subjected to reverse transcription of mRNA using the PrimeScript^TM^ RT Master Mix (TaKaRa, Dalian, China). Reverse transcription reactions for mature miRNAs were conducted with 1 ug total RNA using miRNA specific stem-loop primers (Supplementary Table S11). The reverse transcription products from mRNA and miRNA were used for qPCR using primers listed in *SI Appendix*, Table S11. qPCR was performed using the SYBR Green qPCR mix following the manufacturer’s instructions in a real-time thermal cycler (Bio-Rad, Hercules, CA, USA) using the cycling conditions: 95 °C for 10 min, 40 cycles of 95 °C for 15 s, 60 °C for 30 s and 72 °C for 30 s. Three biological and three technical replicates were performed with expression data analyzed by the 2^−ΔΔCt^ method^49^. Dissociation curves were determined for each mRNA and miRNA to confirm unique amplification. The expression of ribosomal protein 49 (*rp49*) was used as internal controls to normalize the expression of mRNA and the small noncoding RNA U6 was used as internal controls to normalize the expression of miRNA.

### Western blots

Western blots were performed on normal and miR-1-3p mutant female and male using TRA-specific rabbit polyclonal antibody peptide. The TRA antibody epitope sequences are as follows: SKRWRKERHISTDSSSPERYRKYQNNQKKESEIEPSDKTIRRTKTTKPISDDKYAARRNASPSPNYRRKTPEKIPYFVDQVRERDRIRRKYGSTRNKSPPASSKFRRRRSISRSRSRSHSRDSLKTKQRSPARRTNYRRRSISVDREWGGNSKREREREKSRADKDLHGSPRHYQHRSDDRTKNVRRSRSSRTHSRSRTRSRERSSRIGTQNSERHKYRYNENEEQNGN. Rabbit anti-β-Actin polyclonal antibody was utilized as an endogenous control. Total sample proteins were extracted by RIPA lysis Buffer (Frdbio, China), with the quantity determined using the BCA Kit (Frdbio, China). Proteins were subjected to polyacrylamide gel (10%) electrophoresis and transferred onto polyvinylidene difluoride membranes (Millipore, USA). Blocking was performed in 5% (weight per volume [w/v]) skimmed milk at room temperature for 1 h. The membranes were incubated with primary antibodies (Rabbit anti-TRA, 1:1000 and Rabbit anti-β-Actin, 1:5000) in 5% (w/v) skimmed milk at 4 °C overnight. Secondary antibody (Goat anti-Rabbit IgG, 1:5000) (Frdbio, China) was incubated at room temperature for 1 h. Immunological blot detection was performed using an ECL Luminol Substrate Reagent (Frdbio, China).

### Statistical analysis

All experiments were repeated at least three times, except for the small RNA sequencing, and analyzed using GraphPad Prism 5.0 (GraphPad Software, San Diego, CA, USA) or Microsoft Excel (Microsoft, Redmond, WA, USA). Results are expressed as the mean ± SEM. Data was compared with either a two-way ANOVA, with subsequent *t* tests using Bonferroni post-tests for multiple comparisons, or with the Student’s *t* test. For all tests, differences were considered significant when *P* < 0.05.

### Reporting summary

Further information on research design is available in the Nature Research Reporting Summary linked to this article.

## Supplementary information

Supplementary Information

Peer review File

Reporting Summary

Supplementary Data 1

Supplementary Data 2

Supplementary Data 3

## Data Availability

The miRNA-seq data presented in this article are deposited in GEO database: GSE117310. All relevant data are available from the authors. The source data underlying Figs. 1, 3a-d, 4a, b, 5a-d, 6a-d, 7c, 8b-d and 9a, b and Supplementary Figs 2, 4,a, b, 6a-c, 7a, b, 10a, b and 11a, b are provided as a Source Data file.

## Source data

Source Data

## References

[1] Ashman T (2014). Tree of sex: a database of sexual systems. Sci. Data.

[2] Bachtrog D (2014). Sex determination: why so many ways of doing it?. PLOS Biol..

[3] Blackmon H, Ross L, Bachtrog D (2017). Sex determination, sex chromosomes, and karyotype evolution in insects. J. Hered..

[4] Erickson JW, Quintero JJ (2007). Indirect effects of ploidy suggest X chromosome dose, not the X:A ratio, signals sex in *Drosophila*. PLOS Biol..

[5] Gempe T, Beye M (2011). Function and evolution of sex determination mechanisms, genes and pathways in insects. BioEssays.

[6] Vicoso B, Bachtrog D (2013). Reversal of an ancient sex chromosome to an autosome in *Drosophila*. Nature.

[7] Bopp D, Saccone G, Beye M (2014). Sex determination in insects: variations on a common theme. Sex. Dev..

[8] Zhang P, Stankiewicz RL (1998). Y-linked male sterile mutations induced by P element in *Drosophila melanogaster*. Genetics.

[9] Carvalho AB, Dobo BA, Vibranovski MD, Clark AG (2001). Identification of five new genes on the Y chromosome of *Drosophila melanogaster*. Proc. Natl. Acad. Sci. USA.

[10] Sharma A (2017). Male sex in houseflies is determined by *Mdmd*, a paralog of the generic splice factor gene *CWC22*. Science.

[11] Hall AB (2015). A male-determining factor in the mosquito *Aedes aegypti*. Science.

[12] Krzywinska E, Dennison NJ, Lycett GJ, Krzywinski J (2016). A maleness gene in the malaria mosquito *Anopheles gambiae*. Science.

[13] Criscione, F., Qi, Y. & Tu, Z. J. *GUY1* confers complete female lethality and is a strong candidate for a male-determining factor in *Anopheles stephensi*. *eLife* **5**, e19281 (2016). 10.7554/eLife.19281 PMC5061544 27644420

[14] Clarke AR (2005). Invasive phytophagous pests arising through a recent tropical evolutionary radiation: the *Bactrocera dorsalis* complex of fruit flies. Annu. Rev. Entomol..

[15] Pane A, Salvemini M, Bovi PD, Polito C, Saccone G (2002). The *transformer* gene in *Ceratitis capitata* provides a genetic basis for selecting and remembering the sexual fate. Development.

[16] Lagos D, Koukidou M, Savakis C, Komitopoulou K (2007). The *transformer* gene in *Bactrocera oleae*: the genetic switch that determines its sex fate. Insect Mol. Biol..

[17] Schetelig MF, Milano A, Saccone G, Handler AM (2012). Male only progeny in *Anastrepha suspensa* by RNAi-induced sex reversion of chromosomal females. Insect Biochem. Mol. Biol..

[18] Peng W, Zheng W, Handler AM, Zhang H (2015). The role of the *transformer* gene in sex determination and reproduction in the tephritid fruit fly, *Bactrocera dorsalis* (Hendel). Genetica.

[19] Liu, G., Wu, Q., Li, J., Zhang, G. & Wan, F. RNAi-mediated knock-down of *transformer* and *transformer 2* to generate male-only progeny in the oriental fruit fly, *Bactrocera dorsalis* (Hendel). *Plos ONE* **10**, (2015). 10.1371/journal.pone.0128892 PMC4461288 26057559

[20] Pane A, De Simone A, Saccone G, Polito C (2005). Evolutionary conservation of *Ceratitis capitata transformer* gene function. Genetics.

[21] Alphey L (2002). Re-engineering the sterile insect technique. Insect Biochem. Mol. Biol..

[22] Kiuchi T (2014). A single female-specific piRNA is the primary determiner of sex in the silkworm. Nature.

[23] Lewis BP, Shih I, Jonesrhoades MW, Bartel DP, Burge CB (2003). Prediction of mammalian microRNA targets. Cell.

[24] Lewis BP, Burge CB, Bartel DP (2005). Conserved seed pairing, often flanked by adenosines, indicates that thousands of human genes are microRNA targets. Cell.

[25] Ling L, Kokoza V, Zhang C, Aksoy E, Raikhel A (2017). MicroRNA-277 targets *insulin-like peptides 7* and *8* to control lipid metabolism and reproduction in *Aedes aegypti* mosquitoes. Proc. Natl. Acad. Sci. USA.

[26] Zhang Y (2016). microRNA-309 targets the Homeobox gene *SIX4* and controls ovarian development in the mosquito *Aedes aegypti*. Proc. Natl. Acad. Sci. USA.

[27] Carthew RW, Sontheimer EJ (2009). Origins and mechanisms of miRNAs and siRNAs. Cell.

[28] Mccombs SD, Saul SH (1995). Translocation-based genetic sexing system for the oriental fruit fly (Diptera: Tephritidae) based on pupal color dimorphism. Ann. Entomol. Soc. Am..

[29] Geuverink E, Beukeboom LW (2014). Phylogenetic distribution and evolutionary dynamics of the sex determination genes *doublesex* and *transformer* in insects. Sex. Dev..

[30] Verhulst EC, De Zande LJMV, Beukeboom LW (2010). Insect sex determination: it all evolves around *transformer*. Curr. Opin. Genet. Dev..

[31] Schutt C, Nothiger R (2000). Structure, function and evolution of sex-determining systems in Dipteran insects. Development.

[32] Morrow JL, Riegler M, Frommer M, Shearman DCA (2014). Expression patterns of sex-determination genes in single male and female embryos of two *Bactrocera* fruit fly species during early development. Insect Mol. Biol..

[33] Gabrieli P (2010). Sex and the single embryo: early deveopment in the Mediterranean fruit fly, *Ceratitis capitata*. BMC Dev. Biol..

[34] Hashiyama K, Hayashi Y, Kobayashi S (2011). *Drosophila Sex lethal* gene initiates female development in germline progenitors. Science.

[35] Hediger M (2010). Molecular characterization of the key switch F provides a basis for understanding the rapid divergence of the sex-determining pathway in the housefly. Genetics.

[36] Shukla JN, Palli SR (2012). *Doublesex* target genes in the red flour beetle, *Tribolium castaneum*. Sci. Rep..

[37] Gempe Tanja, Hasselmann Martin, Schiøtt Morten, Hause Gerd, Otte Marianne, Beye Martin (2009). Sex Determination in Honeybees: Two Separate Mechanisms Induce and Maintain the Female Pathway. PLoS Biology.

[38] Verhulst EC, Beukeboom LW, De Zande LJMV (2010). Maternal control of haplodiploid sex determination in the wasp Nasonia. Science.

[39] Bushati N, Stark A, Brennecke J, Cohen SM (2008). Temporal reciprocity of miRNAs and their targets during the maternal-to-zygotic transition in *Drosophila*. Curr. Biol..

[40] Kawaoka S (2011). The silkworm W chromosome is a source of female-enriched piRNAs. RNA.

[41] Willhoeft U, Franz G (1996). Identification of the sex-determining region of the *Ceratitis capitata* Y chromosome by deletion mapping. Genetics.

[42] Papanicolaou A (2016). The whole genome sequence of the Mediterranean fruit fly, *Ceratitis capitata* (Wiedemann), reveals insights into the biology and adaptive evolution of a highly invasive pest species. Genome Biol..

[43] Concha C, Scott MJ (2009). Sexual development in *Lucilia cuprina* (Diptera, Calliphoridae) is controlled by the *transformer* gene. Genetics.

[44] Zheng, W., Peng, T., He, W. & Zhang, H. High-throughput sequencing to reveal genes involved in reproduction and development in *Bactrocera dorsalis* (Diptera: Tephritidae). *Plos ONE* **7**, e36463 (2012). 10.1371/journal.pone.0036463 PMC3343016 22570719

[45] Calla B, Geib SM (2015). MicroRNAs in the oriental fruit fly, *Bactrocera dorsalis*: extending Drosophilid miRNA conservation to the Tephritidae. BMC Genomics.

[46] Enright AJ (2003). MicroRNA targets in *Drosophila*. Genome Biol..

[47] Kruger JP, Rehmsmeier M (2006). RNAhybrid: microRNA target prediction easy, fast and flexible. Nucleic Acids. Res..

[48] Handler AM, Harrell RA (2001). Transformation of the Caribbean fruit fly, *Anastrepha suspensa*, with a piggyBac vector marked with polyubiquitin-regulated GFP. Insect Biochem. Mol. Biol..

[49] Livak KJ, Schmittgen TD (2001). Analysis of relative gene expression data using real-time quantitative PCR and the 2^−ΔΔCT^ method. Methods.

