# Supplementary material for: miRNA-1-3p is an early embryonic male sex-determining factor in the Oriental fruit fly Bactrocera dorsalis
Source: Nat Commun. 2020 Feb 18;11:932. doi: 10.1038/s41467-020-14622-4 (PMC7029022; doi:10.1038/s41467-020-14622-4)
Supplement: Supplementary file 1 — Supplementary Information [file 41467_2020_14622_MOESM1_ESM.pdf]

Supplementary Information

miRNA-1-3p is an early embryonic male sex-determining factor in the Oriental fruit fly *Bactrocera dorsalis*

Peng, *et al*

Other supplementary materials for this manuscript include the following:

Data S1 to S2

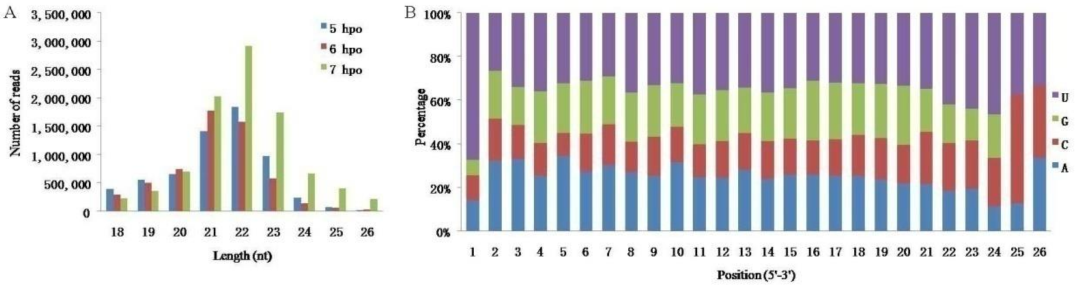

Supplementary Figure 1. The analysis of three small RNA libraries at 5, 6 and 7 hour post-oviposition (hpo).

A) Length distribution and abundance of combined small RNAs in three libraries, and B) first nucleotide bias in different sites from 1 to 26 (5’-3’) in *B. dorsalis* miRNAs.

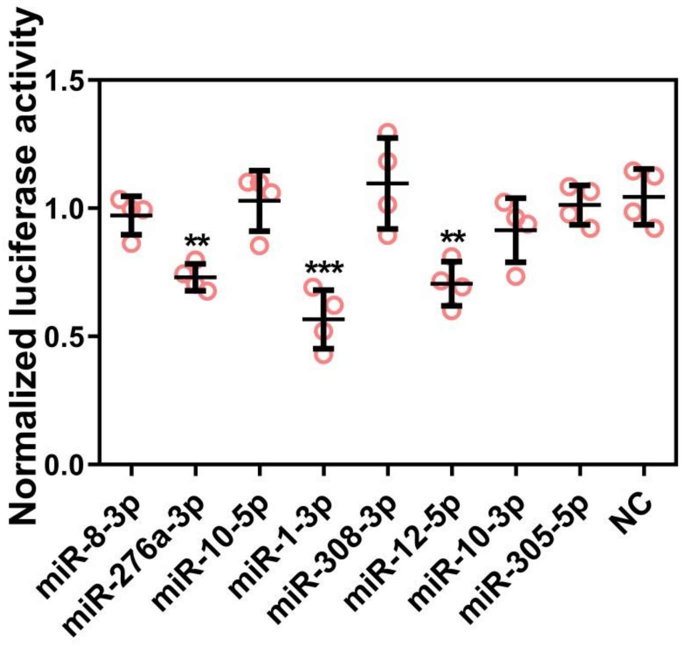

Supplementary Figure 2. Dual-luciferase assay in HEK293T cells.

Error bars indicate the SEM of three independent biological replicates and asterisks (** and ***) indicate the statistically significant differences (*P*<0.01 and *P*<0.001) between the treatment group and control group based on Student's t-test.

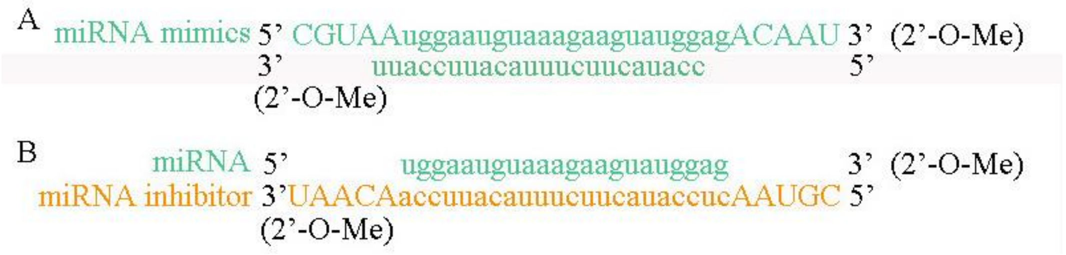

Supplementary Figure 3. The structure of the miR-1-3p agomir (A) and antagomir (B) used in embryonic injections.

The miR-1-3p agomir is a small double-stranded sequence with a chemical modification that results in miR-1-3p overexpression. The miR-1-3p antagomir is an antisense sequence of the mature miR-1-3p with a chemical modification that inhibits endogenous miR-1-3p expression.

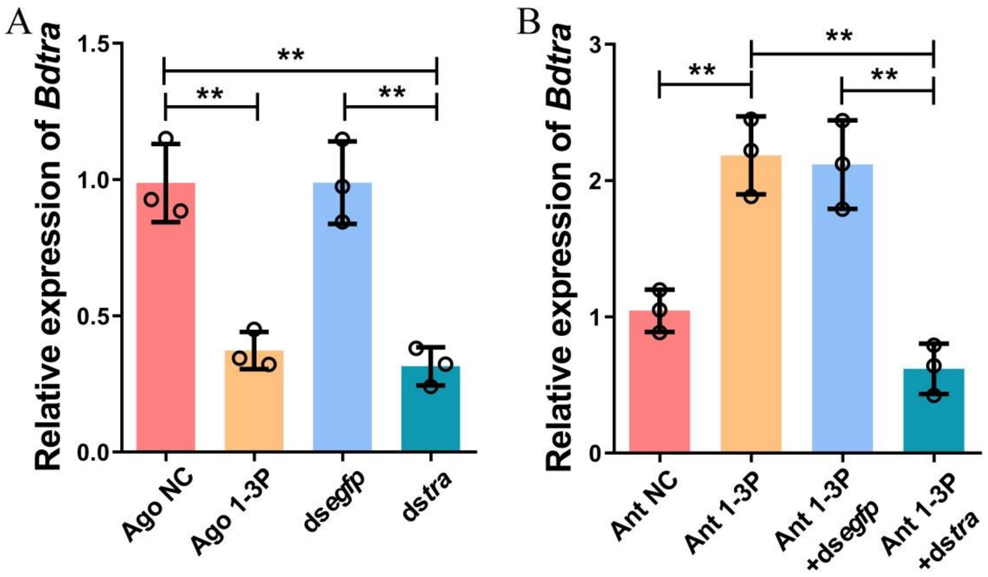

Supplementary Figure 4. (A) Embryonic injection of the miR-1-3p agomir and *transformer* dsRNA (ds*tra)*, and (B) embryonic coinjection of the miR-1-3p antagomir and ds*tra*.

Error bars indicate the SEM of three independent biological replicates and asterisks (** and ***) indicate the statistically significant differences (P<0.01 and P<0.001) between the treatment group and control group based on Student's t-test.

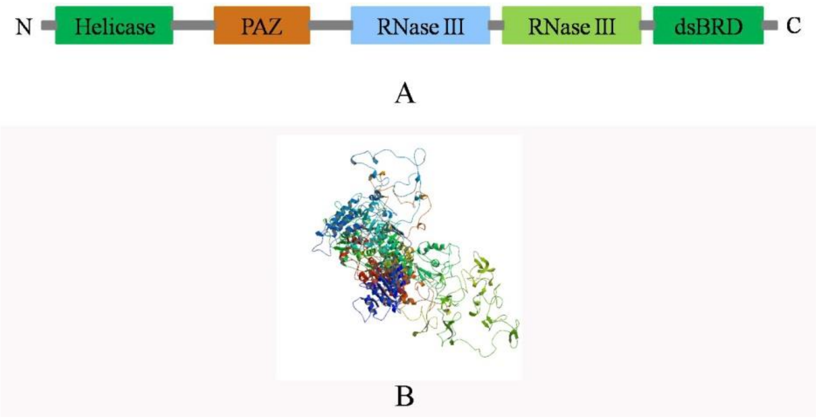

Supplementary Figure 5. The secondary (A) and tertiary (B) structure of Dicer-1 protein in *B. dorsalis*.

Secondary structure of *B. dorsalis* Dicer-1 protein is predicted by Smart (http://smart.embl-heidelberg.de/) and the tertiary structure of *B. dorsalis* Dicer-1 protein is predicted by SWISS-MODEL (https://www.swissmodel.expasy.org/). The Protein Data Bank (PDB) accession code of the template for the tertiary structure is 5ZAL.

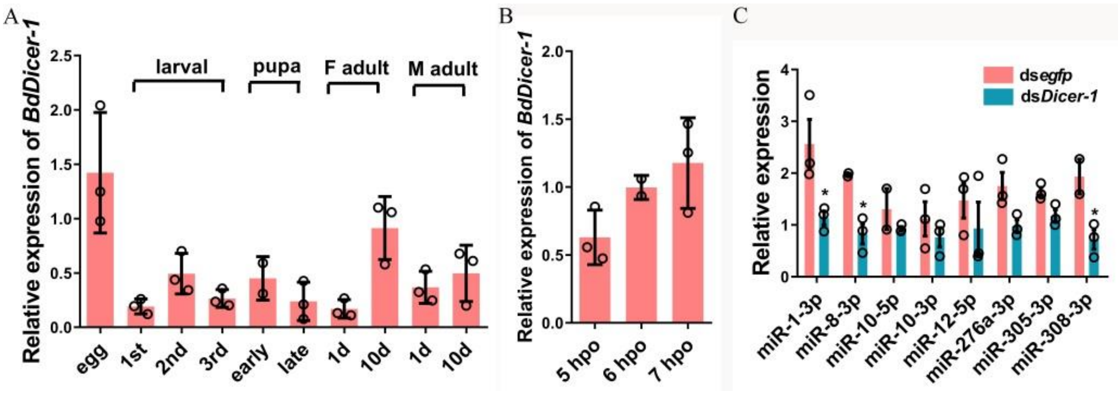

Supplementary Figure 6. (A) qPCR expression profiles of the *BdDicer-1* transcript across different developmental stages, and (B) embryos at 5, 6, 7 hpo, and (C) expression levels of eight miRNAs in embryos injected with ds*BdDicer-1* or ds*egfp*.

Error bars indicate the SEM of three independent biological replicates and asterisks (*) indicate the statistically significant differences (P<0.05) between the treatment group and control group based on Student's t-test.

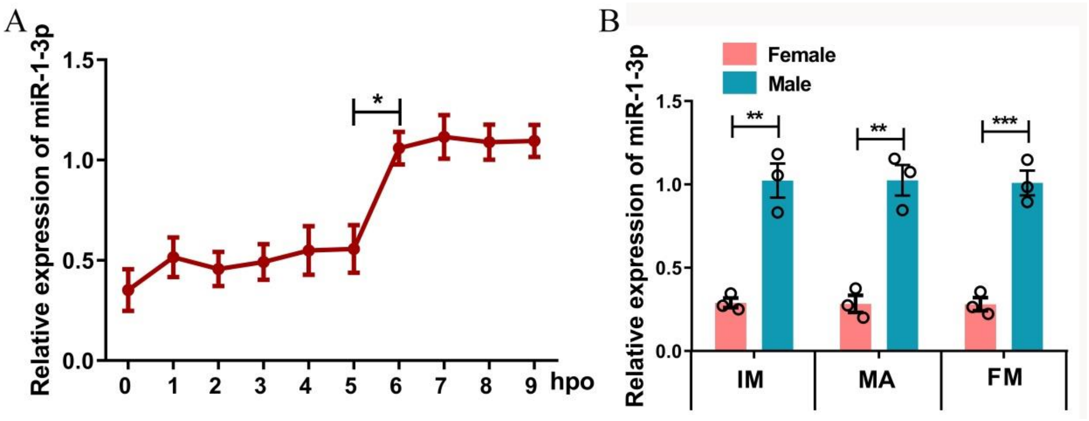

Supplementary Figure 7. (A) Relative levels of miR-1-3p in mixed embryos at 0 to 9 hpo, and (B) in the immature (IM), middle-aged (MA) and fully mature (FM) female and male adults of *B. dorsalis*.

Error bars indicate the SEM of three independent biological replicates and asterisks (* and ** and ***) indicate the statistically significant differences (P<0.05 and P<0.01 and P<0.001) between the treatment group and control group based on Student's t-test.

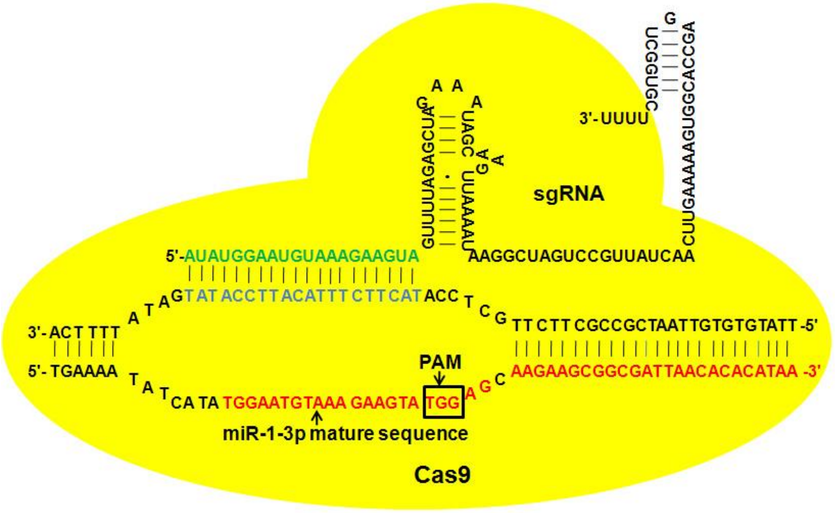

Supplementary Figure 8. A graphical representation of the interaction of miR-1-3p and the miR-1-3p specific sgRNA.

The green sequence is the sgRNA base-pairing region that targets the miR-1-3p mature sequence, proximal to the TGG protospacer-adjacent motif (PAM).

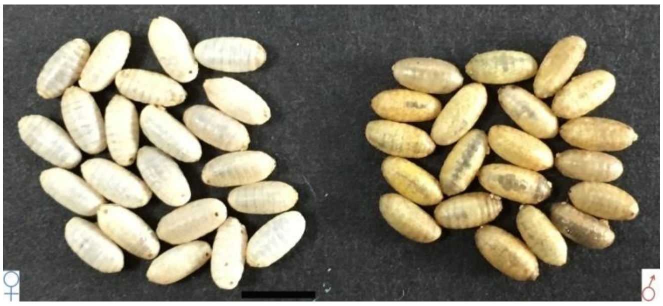

Supplementary Figure 9. Pupae from the white/brown pupae sexing strain showing XX female white pupae (*wp*$^{-}$; left) and XY male brown pupae (*wp*$^{+}$; right).

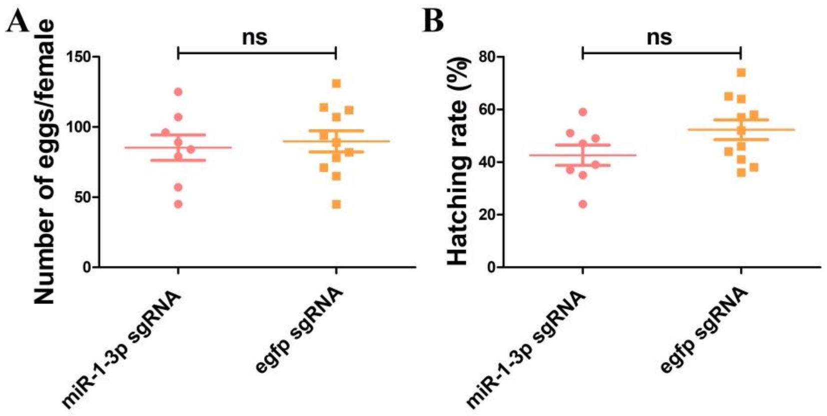

Supplementary Figure 10. The number of eggs oviposited (A) and hatching rate (B) by mutant miR-1-3p females created by the CRISPR/Cas9 system.

Mutant females refer to mutations created by CRISPR/Cas9 using an miR-1-3p sgRNA. Females injected with *egfp* sgRNA females are controls, and Ns indicates no statistically significant difference between treatment group and the control group based on Student's t-test.

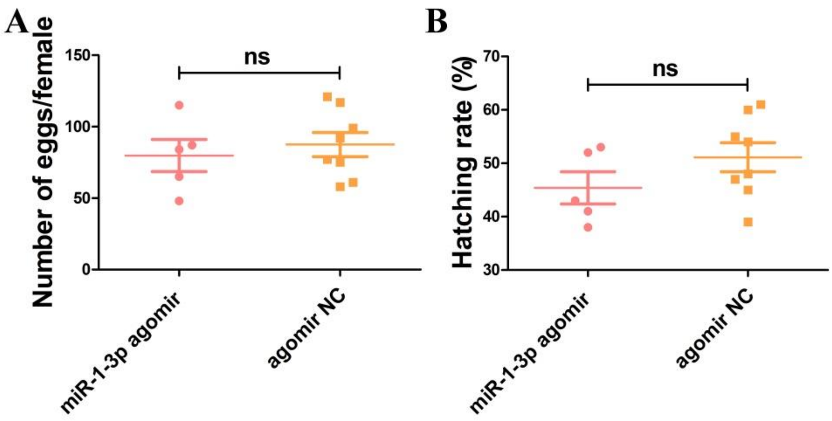

Supplementary Figure 11. The number of eggs oviposited (A) and hatching rate (B) by females mated to masculinized XX males injected as embryos with a miR-1-3p agomir or agomir NC.

Ns indicates no statistically significant difference between the treatment and control groups based on Student's t-test.

Supplementary Table 1.
Classification of sequenced reads in three embryonic small RNA libraries

type	5 hpo		6 hpo		7 hpo	
	Total	%	Total	%	Total	%
Raw reads	9,764,790	100	9,899,586	100	11,795,803	100
3ADT&length filter	3,244,819	33.23	3,995,968	40.37	2,118,104	17.96
Junk reads	17,439	0.18	19,901	0.20	39,283	0.33
Rfam	319,150	3.27	185,311	1.87	380,392	3.22
Repeats	5,014	0.05	2,674	0.03	2,834	0.02
rRNA	247,163	2.53	147,998	1.49	265,970	2.25
tRNA	23,367	0.24	14,947	0.15	66,210	0.56
snoRNA	13,964	0.14	7,879	0.08	8,033	0.07
snRNA	4,664	0.05	2,699	0.03	7,877	0.07
other Rfam RNA	29,992	0.31	11,788	0.12	32,302	0.27
Clean reads	6,178,809	63.28	5,695,996	57.54	9,255,550	78.46

Supplementary Table 2.

Differentially expressed miRNAs at 5, 6 and 7 hpo in *B. dorsalis* embryos

miRNA name	miRNA sequences	P value (chi square nxn)	5 hpo (norm)	6 hpo (norm)	7 hpo (norm)
miR-6-3p	CATCACAGTGGCTGTTCTTT	0.00E+00	156,179	254,134	254,494
miR-286-3p	TGACTAGACCGAACACTCGTGCT	0.00E+00	79,921	101,362	225,001
miR-7-5p	TGGAAGACTAGTGATTTTGTTGTT	0.00E+00	3,855	5,869	21,160
miR-5-5p	AAAGGAACGTTCGTTGTGATAT	0.00E+00	4,728	11,698	20,170
miR-8-3p	TAATACTGTCAGGTAAAGATGTC	0.00E+00	20,798	2,877	17,612
miR-9b-5p	TCTTTGGTGATTTTAGCTGTAT	6.26E-49	8,759	9,331	14,885
miR-9c-5p	TCTTTGGTATTCTAGCTGTAG	1.49E-24	7,571	9,316	10,751
miR-92b-3p	AATTGCACTAGTCCCGGCCTGC	4.65E-67	4,905	5,257	9,133
miR-999-3p	TGTTAACTGTAAGACTGTGTCT	3.56E-80	6,927	8,832	8,854
miR-1-3p	TGGAATGTAAAGAAGTATGGAG	0.00E+00	3,412	272	8,342
miR-10-5p	ACCCTGTAGATCCGAATTTGTT	0.00E+00	3,641	830	7,871
miR-5-3p	TATCACAGTGATTTTCCTTGT	2.34E-17	5,143	6,578	7,761
miR-11-3p	CATCACAGTCTGAGTTCTTGC	0.00E+00	7,832	5,490	5,717
miR-276a-3p	TAGGAACTTCATACCGTGCTCT	0.00E+00	23,089	2,082	5,502
miR-125-5p	TCCCTGAGACCCTAACTTGTGA	0.00E+00	10,597	1,412	4,184
miR-184-3p	TGGACGGAGAACTGATAAGGGC	0.00E+00	4,625	1,399	3,874
miR-956-3p	TTTCGAGACCACTCTAATCCATT	0.00E+00	5,065	393	3,211
miR-14-3p	TCAGTCTTTTTCTCTCTCCTAT	0.00E+00	9,587	1,337	3,153
let-7-5p	TGAGGTAGTAGGTTGTATAGT	0.00E+00	6,826	1,257	3,049
miR-309-3p	TCACTGGGTAAAGTTTGTCCT	2.77E-48	2,344	2,401	2,306
miR-263a-5p	AATGGCACTGGAAGAATTCACGG	3.77E-68	1,908	1,101	2,087
miR-306-5p	CCAGGTACTTAGTGACTCTC	0.00E+00	2,675	3,068	1,743
miR-12-5p	TGAGTATTACATCAGGTACTGGT	0.00E+00	1,563	521	1,679
miR-34-5p	TGGCAGTGTGGTTAGCTGGTT	0.00E+00	1,302	123	832
miR-10-3p	CAAATTCGGTTCTAGAGAGGTTT	0.00E+00	930	59	654
miR-958-3p	TGAGATTCTTCTATTCTACTTT	0.00E+00	1,051	95	591
miR-996-3p	TGACTAGATTTCATGCTCGT	0.00E+00	1,078	662	546
miR-317-3p	TGAACACAGCTGGTGGTATCC	0.00E+00	1,089	306	503
miR-308-3p	TATCACAGGATTATACTG	0.00E+00	1,580	475	446
miR-988-5p	GTGTGATTTGTAGCAAAGTGATT	1.57E-38	58	189	430
miR-9b-3p	TAGAGCTTTATTACCAAAAACC	5.72E-72	49	48	408
miR-281-5p	AAGAGAGCTATCCGTCGACAGTC	0.00E+00	943	67	403
miR-994-3p	TATCACAGTGGCTGTTTCTTAT	2.18E-11	392	455	388
miR-13b-3p	TATCACAGCCATTTTGACGAGTT	7.69E-11	317	189	354
miR-305-5p	ATTGTACTTCATCAGGTGCTC	0.00E+00	859	104	339
miR-184-3p	TGGACGGAGAACTGATAAGGGCA	1.81E-06	222	150	314
miR-318-3p1	TCACTGGGCTTTGTTTATCTC	1.57E-07	180	306	274
miR-9a-5p	TCTTTGGTTATCTAGCTGTATGA	0.00E+00	2,677	279	266
miR-252-5p	CTAAGTACTAGTGCCGCAGGAG	1.85E-59	319	28	255

miR-2b-3p	TATCACAGCCAGCTTTGAGGAG	1.58E-29	317	113	251
miR-989-3p	TGTGATGTGACGTAGTGGAAC	1.37E-08	154	272	224
miR-283-5p	AAATATCAGCTGGTAATTCTG	6.78E-06	150	96	222
miR-970-3p	TCATAAGACACACGCGGCTAT	0.00E+00	444	79	213
miR-281-3p	TGTCATGGAATTGCTCTCTTT	0.00E+00	551	70	195
miR-375-3p	TTTGTTCGTTTGGCTTAAGTT	7.17E-47	280	44	195
miR-994-5p	TAAGGAAATAGTAGCCGTGATT	1.04E-02*	118	183	195
miR-2a-3p	TCACAGCCAGCTTTGATGAGCT	2.96E-59	335	69	177
miR-210-3p	CTTGTGCGTGTGACAGCGGCT	1.45E-28	200	38	162
miR-276b-3p	TAGGAACTTAATACCGTGCTCT	0.00E+00	407	48	156
miR-8-5p	CATCTTACCGGGCAGCATTAGA	0.00E+00	420	49	152
miR-986-5p	TCTCGAATAGCGTTGTGACTGA	0.00E+00	356	41	142
miR-304-5p	TAATCTCAATTTGTAACTGTGA	1.25E-02*	97	72	141
miR-995-3p	TAGCACCACATGATTCGGCTT	5.38E-36	290	136	134
miR-305-3p	CGGCACATGTTGAAGTACACTCA	8.74E-45	229	30	134
miR-981-3p	TTCGTTGTCGACGAAACCTGCA	9.95E-18	101	12	134
miR-34-3p	CAGCCACTATCTTCACTGCCGCC	5.44E-43	186	5	127
miR-124-5p	GGTATCCACTGTAGGCCTATATG	1.09E-29	5	11	127
miR-190-5p	AGATATGTTTGATATTCTTGGTTG	3.57E-13	107	25	119
miR-2b-5p	TTCTTCAAAGCGGTTGTGAAAT	4.39E-05	96	50	119
miR-275-3p	TCAGGTACCTGAAGTAGCGCGCG	5.82E-48	196	8	111
miR-9a-3p	TAAAGCTAGCTTACCGAAGTTA	1.02E-23	126	11	106
miR-1000-5p	ATATTGTCCTGTCACAGCAGT	6.55E-29	125	10	68
miR-31b-5p	TGGCAAGATGTCGGCATAGCTGA	2.65E-35	124	3	56
miR-31a-5p	TGGCAAGATGTCGGCATAGCTGA	2.65E-35	124	3	56
miR-14-5p	GGGAGCGAGACGGGGACTCACT	6.53E-49	136	4	34

* is p value <=0.05, others are p value <=0.001. The statistically significant difference was tested with a Fisher's test and a chi-squared test.

Supplementary Table 3.

Read counts of the eight miRNAs that showed predicted targets in the *transformer* gene 3' UTR

miRNA	5 hpo reads	6 hpo reads	7 hpo reads
miR-8-3p	20798	2877	17612
miR-276a-3p	23089	2082	5502
miR-10-5p	3641	830	7871
**miR-1-3p**	**3412**	**272**	**8342**
miR-308-3p	1580	475	446
miR-12-5p	1563	521	1679
miR-10-3p	930	59	654
miR-305-5p	859	104	339

Supplementary Table 4.

Sex bias induced by embryonic injection of either an miR-1-3p agomir or antagomir in *B. dorsalis*

Injected treatment	Injected embryos	Adults	Male (%)*	Female (%)	Intersex (%)
uninjected	400	313	147 (47%)	166 (53%)	0
Ago-1-3p rep1	427	54	**47 (87%)**	5 (9%)	2 (4%)
Ago-1-3p rep2	406	48	**44 (92%)**	3 (6%)	1 (2%)
Ago-1-3p rep3	412	51	**45 (88%)**	4 (8%)	2 (4%)
Ago NC	408	39	18 (46%)	21 (54%)	0
Ant-1-3p rep1	447	53	14 (26%)	**36 (68%)**	3 (6%)
Ant-1-3p rep2	402	44	9 (21%)	**34 (77%)**	1 (2%)
Ant-1-3p rep3	423	46	13 (28%)	**31 (68%)**	2 (4%)
Ant NC	411	47	22 (47%)	25 (53%)	0

*The percentages in brackets refer to the percentages of each sex among all adults from each treatment.

Supplementary Table 5.

Adult sex ratios after embryonic injection of either the miR-1-3p mutant agomir (ago), antagomir (ant) or NC controls in *B. dorsalis*

Injected treatment	Injected embryos	Adults	Male (%)	Female (%)	Intersex (%)
mutant ago	247	35	17 (49%)	18 (51%)	0
mutant ago NC	230	25	13 (52%)	12 (48%)	0
mutant ant	244	27	15 (56%)	12 (44%)	0
mutant ant NC	251	33	15 (45%)	18 (55%)	0

Supplementary Table 6.
Induced female bias by CRISPR/Cas9-mediated knockout of miR-1-3p

Injected treatment*	Injected embryos	Larvae	Pupae	Adults			Mutants		
				Female (%)	Male (%)	Intersex (%)	Female (%)	Male (%)	Intersex (%)
Cas9 mRNA + miR-1-3p sgRNA	706	118	83	58 (81%)	11 (15%)	3 (4%)	21 (100%)	0	3 (100%)
Cas9 mRNA + *egfp* sgRNA	722	147	104	41 (48%)	44 (52%)	0	0	0	0

*The hatching rate, pupation rate and eclosion rate are similar between the treatment and control groups (not shown, but can be calculated using the data provided in the table). Note that targeting miR-1-3p for knockout resulted in a female sex bias (81%) and 21 of the miR-1-3p mutants are phenotypic females and 3 of the miR-1-3p mutants are intersex individuals.

Supplementary Table 7.

Male to female sex conversion in the *B. dorsalis* white/brown pupae sexing strain by CRISPR/Cas9-mediated knockout of miR-1-3p

Injected treatment*	Injected embryos	Larvae	Pupae			Adults				
			white	brown	Total	white pupae		brown pupae		Total
						Females	males	Females	Males	
Cas9 mRNA + miR-1-3p sgRNA	714	115	16	57	73	15 (3 mutants)	0	11 (11 mutants)	36 (5 mutant)	62
Cas9 mRNA + *egfp* sgRNA	729	121	19	65	84	17 (0 mutant)	0	0	58 (0 mutant)	75

*The hatching rate, pupation rate and eclosion rate are similar between the experimental and control treatment groups (not shown, but can be calculated using the data provided in the table).

Supplementary Table 8.

F1 adult sex ratios in the progeny of feminized XY females

Injected treatment	Male (%)	Female (%)
1	22 (76%)	7 (24%)
2	23 (62%)	14 (38%)
3	14 (50%)	14 (50%)
4	6 (56%)	5 (45%)
CK1	25 (66%)	13 (34%)
CK2	27 (59%)	19 (41%)
CK3	20 (44%)	25 (55%)
CK4	14 (38%)	23 (62%)

Supplementary Table 9.

Female to male conversion in the *B. dorsalis* white/brown pupae sexing strain after embryonic injection of an miR-1-3p agomir

Injected treatment	Injected embryos	Larvae	Pupae			Adults				
			white	brown	Total	white pupae		brown pupae		Total
						Females	males	Females	Males	
Ago-1-3p	684	81	14	42	56	5	**8**	0	39	52
Ago NC	697	86	11	44	55	11	0	0	41	52

The hatching rate, pupation rate and eclosion rate are similar between the treatment and control groups (not shown, but can be calculated using the data provided in the table). Note that miR-1-3p agomir treatments resulted in female to male sex conversion in 8 of the white genetic females.

Supplementary Table 10.

F1 adult sex ratios in progeny of wild type XX females mated to masculinized XX males

Injected treatment	Male (%)	Female (%)
1	3 (11%)	25 (89%)
2	5 (14%)	31 (86%)
3	2 (11%)	16 (89%)
CK1	31 (72%)	12 (28%)
CK2	35 (64%)	20 (36%)
CK3	28 (76%)	9 (24%)

Supplementary Table 11
Oligonucleotide primers

Primer names	Sequence 5’ to 3’
Stem loop primers*:	
miR-1-3p SL	GTCGTATCCAGTGCAGGGTCCGAGGTATTCGCACT GGATACGACCTCCAT
miR-8-3p SL	GTCGTATCCAGTGCAGGGTCCGAGGTATTCGCACT GGATACGACGACATC
miR-10-5p SL	GTCGTATCCAGTGCAGGGTCCGAGGTATTCGCACT GGATACGACAACAAA
miR-10-3p SL	GTCGTATCCAGTGCAGGGTCCGAGGTATTCGCACT GGATACGACAAACCT
miR-12-5p SL	GTCGTATCCAGTGCAGGGTCCGAGGTATTCGCACT GGATACGACACCAGT
miR-276a-3p SL	GTCGTATCCAGTGCAGGGTCCGAGGTATTCGCACT GGATACGACAGAGCA
miR-305-5p SL	GTCGTATCCAGTGCAGGGTCCGAGGTATTCGCACT GGATACGACGAGCAC
miR-308-3p SL	GTCGTATCCAGTGCAGGGTCCGAGGTATTCGCACT GGATACGACCAGTAT
U6 SL	GTCGTATCCAGTGCAGGGTCCGAGGTATTCGCACT GGATACGACACGATTTTGCGT
sub-cloning primers:	
tra 3’ F	CCCTCGAG TAAATTATGGGTAATACACGTAGAT
tra 3’ R	TTGCGGCCGC GCGGATCCTCCACTAGTGATTTCA
qPCR primers:	
miR-1-3p F	TGGAATGTAAAGAAGTATGGAG
miR-8-3p F	TAATACTGTCAGGTAAAGAT
miR-10-5p F	ACCCTGTAGATCCGAATTT
miR-10-3p F	CAAATTCGGTTCTAGAGAGG
miR-12-5p F	TGAGTATTACATCAGGTAC
miR-276a-3p F	TAGGAACTTCATACCGTGCTCT
miR-305-5p F	ATTGTACTTCATCAGGTGCT
miR-308-3p F	TATCACAGGATTATACTG
U6 F	AGGATGACACGCAAAATCGT
General miRNA R	CAGTGCAGGGTCCGAGGTAT
traf F	TTCCAAAACTGTTGGCAACATCAAG
traf R	GAACCTTCACCGAATCTACG
tram F	TTGGCTCAATTAGGAAGGGTC
tram R	GTGCGTCATTTGTGCAAGCT
rp49 F	CCCGTCATATGCTGCCAACT
rp49 R	GCGCGCTCAACAATTTCCTT
dsRNA synthesis primers：	
Dicer-1 F	AGATGCCGTTGTTATGCC

Dicer-1 R	AACTCCGTTGCCTTTCAC
Dicer-1 T7 F	GGATCCTAATACGACTCACTATAGGAGATGCCGTTGTTATGCC
Dicer-1 T7 R	GGATCCTAATACGACTCACTATAGGAACTCCGTTGCCTTTCAC
tra F	AAGTAAGGGAGCGAGACA
tra R	GGCATAGGATAAGTGGGA
tra T7 F	GGATCCTAATACGACTCACTATAGGAAGTAAGGGAGCGAGACA
tra T7 R	GGATCCTAATACGACTCACTATAGGGGCATAGGATAAGTGGGA
egfp F	ACGTAAACGGCCACAAGTTC
egfp R	AAGTCGTGCTGCTTCATGTG
egfp T7 F	GGATCCTAATACGACTCACTATAGGACGTAAACGGCCACAAGTTC
egfp T7 R	GGATCCTAATACGACTCACTATAGGAAGTCGTGCTGCTTCATGTG
Bdtra exon-specific primers：	
Bdtra F	TCCAGACGAAGTTGTTAT
Bdtra R	TTATGATTTGCGGTTGTG
Bdtra and *Bddsx* amplification primers:	
Bdtraf F	TTCCAAAACTGTTGGCAACATCAAG
Bdtraf R	GAACCTTCACCGAATCTACG
Bdtram F	TTGGCTCAATTAGGAAGGGTC
Bdtram R	GTGCGTCATTTGTGCAAGCT
Bddsxf F	TTCTACAATCAACTATCCAATCCG
Bddsxf R	GTGCCCTTTAACTCCAATGC
Bddsxm F	TACTGACTTCGCACTTCTCGCA
Bddsxm R	GAAGGCTTAGATGGACTGGGTG
sgRNA synthesis primers：	
CRISPR forward primer for miR-1-3p sgRNA	GAAATTAATACGACTCACTATAGGATATGGAATGTAAAGAAGTAGTTTTAGAGCTAGAAATAGC
CRISPR forward primer for *egfp* sgRNA	TAATACGACTCACTATAGGGCGAGGAGCTGTTCACCGGTTTTAGAGCTAGAAATAGC
CRISPR Universal reverse primer	AAAAGCACCGACTCGGTGCCACTTTTTCAAGTTGATAACGGACTAGCCTTATTTTAACTTGCTATTTCTAGCTCTAAAAC
somatic mutations primers：	
miR-1-3p F	GTTTAGTTCCATTCTTCCTTGC
miR-1-3p R	TTTAGTCTTCGGTACGCT

Notes: *SL (Stem loop) primers are used for cDNA synthesis of miRNAs; others are used for qPCR, sub-cloning RCR, dsRNA synthesis, sgRNA synthesis and identification of somatic mutations.

Supplementary Table 12.
miRNA agomir and antagomir sequences

miRNA	Sequence 5’ to 3’
agomir	
miR-1-3p sense strand	UGGAAUGUAAAGAAGUAUGGAG
miR-1-3p antisense strand	CCAUACUUCUUUACAUUCCAUU
miR-1-3p mutant sense strand	UGGCCCACACCACCGCCUGACA
miR-1-3p mutant antisense strand	UCAGGCGGUGGUGUGGGCCAUU
miR-8-3p sense strand	UAAUACUGUCAGGUAAAGAUGUC
miR-8-3p antisense strand	CAUCUUUACCUGACAGUAUUAUU
miR-10-5p sense strand	ACCCUGUAGAUCCGAAUUUGUU
miR-10-5p antisense strand	CAAAUUCGGAUCUACAGGGUUU
miR-10-3p sense strand	CAAAUUCGGUUCUAGAGAGGUUU
miR-10-3p antisense strand	ACCUCUCUAGAACCGAAUUUGUU
miR-12-5p sense strand	UGAGUAUUACAUCAGGUACUGGU
miR-12-5p antisense strand	CAGUACCUGAUGUAAUACUCAUU
miR-276a-3p sense strand	UAGGAACUUCAUACCGUGCUCU
miR-276a-3p antisense strand	AGCACGGUAUGAAGUUCCUAUU
miR-305-3p sense strand	AUUGUACUUCAUCAGGUGCUC
miR-305-3p antisense strand	GCACCUGAUGAAGUACAAUUU
miR-308-3p sense strand	UAUCACAGGAUUAUACUG
miR-308-3p antisense strand	GUAUAAUCCUGUGAUAUU
N.C agomir-sense strand	UUCUCCGAACGUGUCACGUTT
N.C agomir-antisense strand	ACGUGACACGUUCGGAGAATT
antagomir	
miR-1-3p	CUCCAUACUUCUUUACAUUCCA
mutant miR-1-3p	UGUCAGGCGGUGGUGUGGGCCA
miR-8-3p	GACAUCUUUACCUGACAGUAUUA
miR-10-5p	AACAAAUUCGGAUCUACAGGGU
miR-10-3p	AAACCUCUCUCGAACCUAAUUUG
miR-12-5p	ACCAGUACCUGAUGUAAUACUCA
miR-276a-3p	AGAGCACGGUAUGAAGUUCCUA
miR-305-3p	UGAGUGUACUUCAACAUGUGCCG
miR-308-3p	CAGUAUAAUCCUGUGAUA
N.C antagomir	UUGUACUACACAAAAGUACUG
