# Supplementary material for: miRNA-1-3p is an early embryonic male sex-determining factor in the Oriental fruit fly Bactrocera dorsalis
Source: Nat Commun. 2020 Feb 18;11:932. doi: 10.1038/s41467-020-14622-4 (PMC7029022; doi:10.1038/s41467-020-14622-4)
Supplement: Supplementary file 3 — Reporting Summary [file 41467_2020_14622_MOESM3_ESM.pdf]

nature research

Corresponding author(s): Hongyu Zhang

Reporting Summary

Nature Research wishes to improve the reproducibility of the work that we publish. This form provides structure for consistency and transparency in reporting. For further information on Nature Research policies, see Authors & Referees and the Editorial Policy Checklist.

Statistical parameters

When statistical analyses are reported, confirm that the following items are present in the relevant location (e.g. figure legend, table legend, main text, or Methods section).

n/a	Confirmed	
☐	☒	The exact sample size (*n*) for each experimental group/condition, given as a discrete number and unit of measurement
☐	☒	An indication of whether measurements were taken from distinct samples or whether the same sample was measured repeatedly
☐	☒	The statistical test(s) used AND whether they are one- or two-sided<br>*Only common tests should be described solely by name; describe more complex techniques in the Methods section.*
☐	☒	A description of all covariates tested
☐	☒	A description of any assumptions or corrections, such as tests of normality and adjustment for multiple comparisons
☐	☒	A full description of the statistics including central tendency (e.g. means) or other basic estimates (e.g. regression coefficient) AND variation (e.g. standard deviation) or associated estimates of uncertainty (e.g. confidence intervals)
☐	☒	For null hypothesis testing, the test statistic (e.g. *F*, *t*, *r*) with confidence intervals, effect sizes, degrees of freedom and *P* value noted<br>*Give P values as exact values whenever suitable.*
☐	☒	For Bayesian analysis, information on the choice of priors and Markov chain Monte Carlo settings
☐	☒	For hierarchical and complex designs, identification of the appropriate level for tests and full reporting of outcomes
☐	☒	Estimates of effect sizes (e.g. Cohen's *d*, Pearson's *r*), indicating how they were calculated
☐	☒	Clearly defined error bars<br>*State explicitly what error bars represent (e.g. SD, SE, CI)*

Our web collection on statistics for biologists may be useful.

Software and code

Policy information about availability of computer code

Data collection	no software was used.
Data analysis	RNAfold software (http://rna.tbi.univie.ac. at/cgi-bin/RNAfold.cgi) for microRNA hairpin RNA structures;<br>miRanda, RNAhybrid, and TargetScan for the prediction of miRNAs target gene;<br>the CasOT search tool for potential off-target binding;<br>GraphPad Prism 5.0 (GraphPad Software, La Jolla, CA, USA) or Microsoft Excel (Microsoft, Redmond, WA, USA) for the graphs making.

For manuscripts utilizing custom algorithms or software that are central to the research but not yet described in published literature, software must be made available to editors/reviewers upon request. We strongly encourage code deposition in a community repository (e.g. GitHub). See the Nature Research guidelines for submitting code & software for further information.

Data

Policy information about availability of data

All manuscripts must include a data availability statement. This statement should provide the following information, where applicable:

- Accession codes, unique identifiers, or web links for publicly available datasets
- A list of figures that have associated raw data
- A description of any restrictions on data availability

The miRNA-seq data presented in this article is deposited in GEO database: GSE117310.

Field-specific reporting

Please select the best fit for your research. If you are not sure, read the appropriate sections before making your selection.

☒ Life sciences ☐ Behavioural & social sciences ☐ Ecological, evolutionary & environmental sciences

For a reference copy of the document with all sections, see nature.com/authors/policies/ReportingSummary-flat.pdf

Life sciences study design

All studies must disclose on these points even when the disclosure is negative.

Sample size	no sample-size calculation was performed.
Data exclusions	no data were excluded from the analysed.
Replication	all attempts at replication were successful.
Randomization	This is not relevant to our study, because we compared samples with different treatments.
Blinding	This is not relevant to our study, because samples were not allocated to groups.

Reporting for specific materials, systems and methods

Materials & experimental systems

n/a	Involved in the study	
☒	☐	Unique biological materials
☐	☒	Antibodies
☐	☒	Eukaryotic cell lines
☒	☐	Palaeontology
☐	☒	Animals and other organisms
☐	☒	Human research participants

Methods

n/a	Involved in the study	
☒	☐	ChIP-seq
☒	☐	Flow cytometry
☒	☐	MRI-based neuroimaging

Antibodies

Antibodies used	Rabbit anti-β-Actin polyclonal antibody; Rabbit anti-TRA, 1:1000; Rabbit anti-β-Actin, 1:5000; Goat anti-Rabbit IgG, 1:5000.
Validation	Antibodies were already validated by the research community.

Eukaryotic cell lines

Policy information about cell lines

Cell line source(s)	HEK293T cell line from our lab.
Authentication	HEK293T cell line was authenticated by the research community.

Mycoplasma contamination	HEK293T cell line was tested negative for mycoplasma contamination.
Commonly misidentified lines (See ICLAC register)	no commonly misidentified cell lines used in the study.

Animals and other organisms

Policy information about studies involving animals; ARRIVE guidelines recommended for reporting animal research

Laboratory animals	We used Bactrocera dorsalis laboratory stocks. Adults, larvae, pupae and embryos were analyzed.
Wild animals	the study did not involve wild animals.
Field-collected samples	the study did not involve samples collected from the field.

Human research participants

Policy information about studies involving human research participants

Population characteristics	The study did not involve human research participants.
Recruitment	no participants were recruited.
