# Supplementary material for: miRNA-1-3p is an early embryonic male sex-determining factor in the Oriental fruit fly Bactrocera dorsalis
Source: Nat Commun. 2020 Feb 18;11:932. doi: 10.1038/s41467-020-14622-4 (PMC7029022; doi:10.1038/s41467-020-14622-4)
Supplement: Supplementary file 4 — Supplementary Data 1 [file 41467_2020_14622_MOESM4_ESM.doc]

**Description of Additional Supplementary Files**

File Name: Supplementary Data 1
Description: Identification of known miRNAs in three embryonic small RNA libraries

File Name: Supplementary Data 2
Description: Identification of combined novel miRNAs in three embryonic small RNA libraries
