# Supplementary material for: miRNA-1-3p is an early embryonic male sex-determining factor in the Oriental fruit fly Bactrocera dorsalis
Source: Nat Commun. 2020 Feb 18;11:932. doi: 10.1038/s41467-020-14622-4 (PMC7029022; doi:10.1038/s41467-020-14622-4)
Supplement: Supplementary file 2 — Peer review File [file 41467_2020_14622_MOESM2_ESM.pdf]

Reviewers' comments:

Reviewer #1 (Remarks to the Author):

This paper presents an important advance in understanding the remarkable variety of genetic circuits that control sex determination. The authors convincingly demonstrate that the micro RNA miR-1-3p is a major player determining sex for the oriental fruit fly, and that it does so by reducing the level of the transcript from the master, positively-autoregulating sex-switch gene transformer (tra) in this species. Importantly, miR-1-3p is both necessary AND sufficient for male development. The authors show that miR-1-3p acts very early in embryogenesis to determine sex. Since miR-1-3p is shown to be autosomal, it cannot itself be the primary masculinizing signal from the male-determining Y chromosome of this species, but its early regulation of tra suggests that it is likely to be an immediate target of the Y signal and, as such, may lead to the identification of the primary signal itself. Manipulating miR-1-3p can generate remarkably complete sex transformations in either direction. Only one other example of a small RNA's key involvement in sex determination has been reported (piRNA in the silk moth). Interestingly in that case the RNA is actually the primary sex-determining signal arising from the heteromorphic sex chromosome (W) but its job is to feminize the animals, contrary to the masculinizing role of miR-1-3p. Moreover, the silk moth doesn't use a tra ortholog. These results are significant and should be of interest to a wide audience. There are, however, some problems with the paper that need to be addressed.

Major points:
(1) In addition to fairly numerous typos (e.g. 781: ration), there are serious problems with the grammar in this manuscript in many places that will need to be fixed (e.g. 191-194). Perhaps the Nature editorial staff will help the authors out. The authors misuse "which" repeatedly and have consistent problems with plurals.

(2) The authors need to give the reader some idea of what degree of development corresponds to the "hours after oviposition" that they describe. Is the embryo cellularlized at the time of injections, and if so, how do the various injected materials (micro-RNAs, RNAi, CRISPR) get to their targets?

(3) In any presentation of sex transformation results, understanding the nature of those transformations, and the degree to which they are or are not complete, is essential. This is particularly true in this case, since tra is known to positively autoregulate. The authors give the reader no description of sexual dimorphism to help us understand their criteria for sex transformation. They say that some of the transformations allow fertility, yet they do not list what fraction of the transformed animals ARE fertile. They say that incomplete sex transformation are observed (mainly, it appears, as abnormalities in ovaries), but their description of intersexuality is inadequate. Clearly intersexuality is expected, since none of the the various treatments described that masculinize or feminize the animals affect all the target animals. Moreover it seem unlikely that the CRISPER/CAS9 modification of target genes would modify those alleles in EVERY potentially dimorphic cell. If sexual differentiation in this species is cell autonomous, in light of what is known about tra, one would expect the authors to have observed mosaic intersexuality among at least some of the treated progeny, and not true (cellular) intersexuality. The specific nature of intersexuality can be one of the most useful pieces of information for understanding the operation of the sex-determination circuitry. The minimal description the authors provide in Fig. 5 of supposed incomplete feminization (no mention is made of incomplete masculinization, by the way) is inadequate (indeed it does NOT look like mosaic intersexuality, since the one picture of the supposed abnormal sexual phenotype is actually symmetric). Were the genetalia mosaic, and if so, how? In presenting their data, the authors must distinguish complete sex transformation from incomplete sex transformation, and they must provide a more scholarly and convincing description AND discussion of intersexuality (or account for its general absence). If they remove the superfluous information that they have included in this manuscript, they should have no problem doing so within space constraints.

(4) We are told that feminized XY flies are fertile, but the authors give no data on the F1 sex ratios. When mated to wildtype males, was the expected skewing in sex ratio (and progeny death in the case of YY progeny) observed? Analysis of the progeny would be the most convincing way to show that the authors are indeed dealing with sex-reversed XY animals and should have been reported. Since the authors say that they did the matings, why do they not tell us the results! We also need to know whether sex-reversed XX males were also fertile (much less whether any analysis of THEIR progeny had been done) -- or NOT. This information must be included in the manuscript.

Less important points:

(5) Lines 31 & 32: "up to 92% (77%)" may be fine for advertisements, but it is not sufficiently precise for a Scientific article. What does "up to" even mean here? Just state the result.

(6) The experiment described in the paragraph starting on line 231 using a means (the white/brown Y-linked pupa marker) to establish chromosomal sex reversals seems clearly adequate to establish the relevant conclusions. Hence there would seem to be no reason to include the other, more indirect, presumably preliminary experiment (the paragraph beginning at 214 and described in Fig. 6).

(7) While tra has (finally) been found to be is present in a number of higher insects and to be involved in their sex determination, it is misleading (line 272) to characterize it as being "widely conserved" since it evolves so rapidly as to make the orthologs very difficult to identify in even closely related species.

(8) Line 295: It is not necessary to review the functions of various miRNAs. Suffice it to say what is in line 294. Nobody doubts miRNAs are functionally important -- these other functions are irrelevant to the current paper.

(9) Fig 3: why refer to "expression levels" rather than simply "levels"?

(10) Fig 5: Line 753. The figure legend should clearly state WHAT was injected when injections are first mentioned, not first in the second part of the figure.

Fig. 6 (although this figure and its associated text is unnecessary; Fig. 7 suffices). Panel A is unnecessary -- the mode of action of CRISPR/Cas9 is well known. The protospacer and PAM sequences targeted should simply be included in M&M. If this figure does end up in the final manuscript, the authors must indicate that the individuals in red are the PRESUMED feminized males based on an expectation of 1:1 sex ratio (actually it is unclear why the authors didn't estimate 25 rather than 24, since only 25 would have given a 1:1 sex ratio; moreover the figure would be more clear if the estimated number of transformed chromosomal males was indicated IN the red bar, with the number of estimated TRUE females indicated IN the white bar).

Reviewer #2 (Remarks to the Author):

Sex determination mechanisms are bewilderingly diverse and we are only at the beginning of uncovering the various genes involved. Sex is typically determined by a cascade of genes that is conserved at the bottom but undergoes frequent turnover at the top (primary signals). This is particularly true for the male-determining genes in insects. Four such genes have been described in

the last few years from flies and mosquitoes and they are all non-homologous. Intriguingly, although they all induce the splicing of transformer and doublesex in the embryonic stage into the male mode, they may do so in different ways. Peng et al report the identification of a paternally inherited micro-RNA that functions as an (intermediate) male determiner. Although it is not the primary signal (Y-linked M factor) it identifies yet another type of regulation of sex is determination in dipteran insects. This is an interesting finding that adds to our knowledge of the complexity and evolutionary lability of the sex determination process.

The presented functional analyses with knock-down and induction studies are sound and the results are well-presented. Nevertheless, there are some concerns that need to be addressed, or at least be acknowledged by the authors. Why was the focus on embryonic stages 6-9 hours? Why did the authors not consider identifying the M factor in this study? They appear to have all the material (embryonic transcripts) so why not looking at earlier embryonic timepoints?

The fact that the miR-1-3p is expressed in both males and females rightfully led the authors to conclude that it cannot be Y-linked. However, the conclusion that it is autosomal (e.g. line 31) appears premature. Can they exclude that it is X-linked? Why did they not attempt to identify it in the genome sequence (https://www.ncbi.nlm.nih.gov/assembly/GCF_000789215.1)? If this was not possible, they should at least mention this in the text. Can they exclude the existence of multiple copies, one of which is on the Y, and transmitted via sperm to eggs?

The interpretation of the data also requires consideration. It is shown that miR-1-3p is expressed more in embryos that were assigned to be XY because these embryos express male tra mRNA. In principle this could be caused by this tra splicing. In XX individuals (assigned XX because of the presence of female tra mRNA) there is less miR-1-3p, that, in principle, could be caused by TRA. So if miR-1-3p lowers tra transcription, it needs to be shown that tra expression in males is lower than in females. This may be reflected in the injection experiments, but it should be mentioned. The action of miR-1-3p is then likely stabilisation. The target sites described in 133-135 (UTR of a gene?) do not reveal if the target is male or female specific (probably not specific). Why not CRISPR the target sites of tra? Is the maternally provided tra mRNA also degraded?

The Introduction lacks coherence and the different paragraphs are unbalanced. The first paragraph starts with mentioning the diversity of sex determination mechanisms but then strongly focuses on details of the Drosophila sex determination cascade, which is just one example, and an exceptional one, that is not related much to the study presented as it does not have an M factor. The second paragraph lacks a closing sentence. Focussing the introduction on species that do have M would be more appropriate. What was the purpose of the study (cf line 92)? To determine the crucial sex determining window, but then the miRNA was accidentally hit upon? If so, present it that way.

There are a number of inaccurate or in appropriate statements throughout the manuscript:
- In the title and rest of the text the term "male-biased" is used. The term is not entirely correct as the miRNA works before sex determination, i.e. it is not male-biased but male-inducing. The term is also confusing with other genomic studies that compare gene expression levels between the sexes.
- Why calling the M-factor "MDF" as M-factor is typically used in the literature?
- The piRNAs do not act on tra, and are therefore also inappropriately placed in the intro. In addition they are feminizing not masculinizing.
- Throughout the paper "splicing of tra" or any other gene is used, when the splicing of the pre-mRNA (primary transcript) is meant.
For more specific terminology see below under minor comments

There are a number of methodological issues:
- The first experiment described in lines 104-115 is not included in the M&M or is it line 441-442 (which would be in the wrong spot of the M&M)? Why only take 5-7h for this screen? Taking earlier time points would have eliminated the uncertainty whether the 5h amplification is still maternal

provision of tra mRNA.
- N=1 per category is tricky for mixed-sex small RNA datasets, N=3 per non-mixed category is on the lower acceptable end for quantitative analyses according to the LGTC.
- Supplementary table 2 shows the normalized abundance of particular miRNAs and this is the same data that was used for the tests (line 130) that showed that these increased significantly at hpo 7 compared to 5 and 6. How does this work? As in many cases the hpo 7 value is lower than the other two categories. The amount of reads after filtering is not noticeably different between the 3 libraries.
- Why only look at miRNA when the only (?) other known small RNA sex determination factor is a piRNA in Bombyx?
- miR-276a-3p and miR12-5p were suggested from the luciferase assay to target tra, yet these fell of the candidate list at the in vivo test. Could they still be involved, but in example earlier than the measured 10h post-injection time point? Have they been tested in single-sex samples to examine whether they are male-specific?
- Line 167: where does the identification of Dicer-1 in B. dorsalis come from? Predicted annotation from genome assembly? Why are the different sample categories of supplementary figure 6A not mentioned in the M&M?

Minor comments
Line 23: what is "male determination"?
Line 27: what is "a male-biased RNA"?
Line 37: what is "and intermediate male determiner"?
Lines 59-60: this is not entirely correctly phrased: Mdmd prevents the zygotic activation of the tra loop, but not activation of tra itself
Lines 53-61: The recently discovered M in Musca is inaccurately described. Mentioning the CWC22 paralog would be appropriate. M in Musca is not necessarily Y-linked. The word "similarly" in line 61 is inappropriate.
Lines 60-61: remove once "splicing", moreover, genes are not spliced, the transcropts are
Line 65: "none have yet to be identified" means that they have all been identified!
Lines 70-76: difficult to read, split up into two sentences. This sentence also contains inaccuracies
Line 80: SIT is not previously introduced and not well integrated in the text here
Line 86: should "downstream factors" not be "upstream factors" in the cascade?
Line 94: what is meant with "a male-biased expression", better: is expressed only in male embryos? Also "Line 195-196: is male-biased expressed in the male embryo"
Lines 104-114: Looks like the sex of the embryo is determined by assessing the tra spliceforms. It is a bit misleading to say that male spliceforms occur in XY embryo's, if the labelling of XY is derived from the identification of such a spliceform.
Line 116: why do the authors expect such RNAs to be present?
Line 131: How do you know the miRNAs are autosomal?
Line 192-193: check phrasing (English)
Line 194: patter = pattern
Line 272-273: replace "cases" by "orders" and delete last part of sentence
Lines 290-297: these seem results that are not described in the result-section itself. Here it just distracts from the discussion on candidate primary signals in sex determination.
Line 296: delete "which"
Line 322 and throughout the manuscript: The use of "while". Strictly speaking, "while "is used to refer to time, so "whereas" would be the better term
Line 337: the term "M factor" is introduced here for the first time, needs explanation
Line 356 hints to the use of this miRNA in other tephritid species. Is it known to be conserved in other model systems of this family?
Line 366-367: why is the sex ratio of the white pupae strain different from 1:1?
Line 500: "all experiments were repeated at least 3 times" The small RNA sequencing was presumably not. Are the other sample sizes mentioned in the manuscript the cumulative number of all these experiments, or of the larger main experiment (versus non-described pilot studies)?

Reviewer #3 (Remarks to the Author):

This manuscript by Peng et al. describes a role for miR-1-3p in male sex determination in the oriental fruit fly, Bactrocera dorsalis. Key experiments are presented at the end of the paper showing that CRISPR-based mutation of miR-1 leads to sex reversal of XY individuals and, conversely, that miR-1 agomir injections leads to sex reversal of XX individuals. These key experiments are complemented by expression and molecular analyses supporting the regulation Bdtra by miR-1. Overall, the experiments are technically sound and properly controlled, the results are clearly presented and appropriately interpreted and, for the most part, the text is well written. However, there are a few concerns, outlined below, that should be addressed to strengthen the manuscript.

(1) The conclusion that sex determination occurs between 6 and 7 hpo does not seem supported by the data. Sex specific splicing is detected at 6 hpo, indicating that the process of sex determination has already begun at this time.

(2) miR-1 expression level should be shown prior to 6hpo in Fig 4C. Presumably there should be a sexually dimorphic switch in miR-1 expression that leads to sex-specific splicing of tra at 6 hpo. If miR-1 expression is the same at 5 hpo (or 4 hpo) as it is at 6hpo, then it cannot be the presumed trigger that causes the switch in tra splicing.

(3) Ideally, the authors would support the effect of miR-1 on tra with Western blots of Tra protein levels in wildtype and miR-1 mutant males and females.

(4) The authors should show that injection of a miR-1-insensitive version of the tra transcript containing a mutation in the putative miR-1 binding site leads to sex reversal of XY individuals. This does not seem like a difficult experiment, and would provide significant additional support for the model. In addition, the authors could consider analyzing the effects of CRISPR-based mutations in the miR-1 binding site of the tra 3'UTR.

(5) One issue that needs to more fully addressed is how the authors reconcile the sexually dimorphic role of miR-1 in Bactrocera dorsalis with its presumably non-dimorphic role in muscle. Is the sexually dimorphic expression that the authors describe in Fig 4C specific to early embryogenesis? Is miR-1 expressed at equivalent levels in males and females at later stages? The authors should show a broader temporal profile of miR-1 expression to address these issues.

(6) I'm not sure that it is appropriate to conclude that miR-8-3p, miR-10-5p, miR-308-3pm, miR-10-3p and miR-305-5p don't regulate Bdtra in human cells (lines 148-149). Isn't it possible that orthologues of these miRNAs are endogenously expressed in HEK293T, leading to luciferase repression in the absence of the mimics?

(7) There are some issues with the writing. Awkward sentences include those on lines 71-76, lines 107-109, and lines 191-194.

Minor Comments

(1) Line 95: miR-1 acts to regulate Bdtra transcription? The authors haven't shown this, but have shown an effect on Bdtra transcript level and splicing.

Reviewers' comments:

Reviewer #1 (Remarks to the Author):

This paper presents an important advance in understanding the remarkable variety of genetic circuits that control sex determination. The authors convincingly demonstrate that the microRNA miR-1-3p is a major player determining sex for the oriental fruit fly, and that it does so by reducing the level of the transcript from the master, positively-autoregulating sex-switch gene *transformer* (*tra*) in this species. Importantly, miR-1-3p is both necessary AND sufficient for male development. The authors show that miR-1-3p acts very early in embryogenesis to determine sex. Since miR-1-3p is shown to be autosomal, it cannot itself be the primary masculinizing signal from the male-determining Y chromosome of this species, but its early regulation of *tra* suggests that it is likely to be an immediate target of the Y signal and, as such, may lead to the identification of the primary signal itself. Manipulating miR-1-3p can generate remarkably complete sex transformations in either direction. Only one other example of a small RNA's key involvement in sex determination has been reported (piRNA in the silk moth). Interestingly in that case the RNA is actually the primary sex-determining signal arising from the heteromorphic sex chromosome (W) but its job is to feminize the animals, contrary to the masculinizing role of miR-1-3p. Moreover, the silk moth doesn't use a *tra* ortholog. These results are significant and should be of interest to a wide audience. There are, however, some problems with the paper that need to be addressed.

Major points:
(1) In addition to fairly numerous typos (e.g. 781: ration), there are serious problems with the grammar in this manuscript in many places that will need to be fixed (e.g. 191-194). Perhaps the Nature editorial staff will help the authors out. The authors misuse "which" repeatedly and have consistent problems with plurals.
Answer: Thank you for your kind suggestions. We have changed the word "ration" into "ratio". We have revised the sentence "As the sex-specific splicing of *Bdtra* in single embryo which *Bdtra-m* only existed in the XY embryo, we identified the sex of single female and male embryo at 6, 7, 8 and 9 hpo (Fig. 4A and 4B)" into "Based on the detection of only *Bdtra-m* in a single XY embryo, which is the major characteristic of sex-specific splicing of *Bdtra* pre-mRNA, we identified the sex of individual female and male embryos at 6, 7, 8 and 9 hpo (Fig. 4A and 4B)". We have checked and corrected the misuse of "which" and the consistent problems with plurals and other grammar problems in our manuscript.

(2) The authors need to give the reader some idea of what degree of development corresponds to the "hours after oviposition" that they describe. Is the embryo cellularlized at the time of injections, and if so, how do the various injected materials (micro-RNAs, RNAi, CRISPR) get to their targets?
Answer: Thank you for your kind suggestions. In tephritid fruit flies, the

cellularization occurs at 9-12 h after egg layingin *Ceratitis capitata* and 7.5-9 h after egg laying in *Bactrocera tryoni* and *Bactrocera jarvisi* (Gabrieli et al., 2010 and Morrow et al., 2014). Most likely also *B. dorsalis* has similar stage. However, the time of embryo injected in our study is in one hour after egg laying and the embryo should not be cellularlized at this time. So the various injected materials (micro-RNAs, RNAi, CRISPR) can get to their targets.

(3) In any presentation of sex transformation results, understanding the nature of those transformations, and the degree to which they are or are not complete, is essential. This is particularly true in this case, since *tra* is known to positively autoregulate. The authors give the reader no description of sexual dimorphism to help us understand their criteria for sex transformation. They say that some of the transformations allow fertility, yet they do not list what fraction of the transformed animals ARE fertile.
Answer: In present revised manuscript, we have added the description of intersexuality (Line 220-224) and fertility results of sex-reversed XY females and XX males (Line 269-273 and Line 290-294, Supplementary Figs. 10 and 11）in the manuscript as you suggested. Thank you. Fertility tests showed that eight of the 11 feminized XY females (73%) mated normally, and both the number of eggs per female and their hatching rate were similar to the control injected (*egfp* sgRNA/Cas9) females (Supplementary Fig. 10) and five of the 8 sex-reversed XX males (63%) mated normally to normal XX females, both the number of eggs per females and their hatching rate were similar to the control injected (agomir NC) treatment (Supplementary Fig. 11).

They say that incomplete sex transformation are observed (mainly, it appears, as abnormalities in ovaries), but their description of intersexuality is inadequate. Clearly intersexuality is expected, since none of the various treatments described that masculinize or feminize the animals affect all the target animals. Moreover it seem unlikely that the CRISPER/CAS9 modification of target genes would modify those alleles in EVERY potentially dimorphic cell. If sexual differentiation in this species is cell autonomous, in light of what is known about *tra*, one would expect the authors to have observed mosaic intersexuality among at least some of the treated progeny, and not true (cellular) intersexuality. The specific nature of intersexuality can be one of the most useful pieces of information for understanding the operation of the sex-determination circuitry. The minimal description the authors provide in Fig. 5 of supposed incomplete feminization (no mention is made of incomplete masculinization, by the way) is inadequate (indeed it does NOT look like mosaic intersexuality, since the one picture of the supposed abnormal sexual phenotype is actually symmetric). Were the genetalia mosaic, and if so, how? In presenting their data, the authors must distinguish complete sex transformation from incomplete sex transformation, and they must provide a more scholarly and convincing description AND discussion of intersexuality (or account for its general absence). If they remove the superfluous information that they have included in this manuscript, they should have no problem doing so within space constraints.

Answer: In present revised manuscript, we have distinguished the complete sex transformation from incomplete sex transformation, and added the description of intersexuality in the manuscript as you suggested (Line 220-224). In the agomir-1-3p injected treatments, we have observed the incomplete masculinization which intersex individuals exhibited a female dorsal phenotype lacking an ovipositor (Fig. 5A and 5B). In the antagomir-1-3p and miR-1-3p sgRNA injected treatments, we have observed the incomplete feminization which intersex individuals showed a male dorsal phenotype by the absence of bristles on the side of the third tergum (Fig. 5C and 5D). The incompletely feminized intersexual individuals had immature ovaries with non-vitellogenic oocytes, whereas the normal females had normal vitellogenic ovaries with mature oocytes (Figure 6C and 6D). Thank you.

(4) We are told that feminized XY flies are fertile, but the authors give no data on the F1 sex ratios. When mated to wild type males, was the expected skewing in sex ratio (and progeny death in the case of YY progeny) observed? Analysis of the progeny would be the most convincing way to show that the authors are indeed dealing with sex-reversed XY animals and should have been reported. Since the authors say that they did the matings, why do they not tell us the results! We also need to know whether sex-reversed XX males were also fertile (much less whether any analysis of THEIR progeny had been done) -- or NOT. This information must be included in the manuscript.

Answer: Thank you for your kind suggestions. We have done the backcross experiments of feminized XY females and masculinized XX males, which mated to the normal XY males and normal XX females respectively. The results have been incorporated into the body of the paper (Line 269-273 and Line 290-294).

The backcross experiments of feminized XY females, which mated to the normal XY males, showed that eight of the 11 feminized XY females (73%) mated normally, both the number of eggs per female and their hatching rate were similar to the control injected (*egfp* sgRNA/Cas9) females (Supplementary Fig. 10) and the F1 adult sex ratios had no significant bias to either sex (Supplementary Table 8). The total number of progeny was less than CK group, which may result from some YY progeny death (Supplementary Table 8).

The backcross experiments of masculinized XX males, which mated to the normal XX females, showed that five of the 8 masculinized XX males (63%) mated normally, both the number of eggs per female and their hatching rate were similar to the control injected (agomir NC) treatment (Supplementary Fig. 11) and the dominant of F1 progeny were females maybe due to Y chromosome’s lack (Supplementary Table 10).

Less important points:

(5) Lines 31 & 32: "up to 92% (77%)" may be fine for advertisements, but it is not sufficiently precise for a Scientific article. What does "up to" even mean here? Just state the result.

Answer: Thank you for your kind suggestions. “up to” means “reach up to”， which is the most highest rate of the progeny. We have modified this sentence into “Injection

of a miR-1-3p mimic in early embryo resulted in 87-92% phenotypic males, whereas knockdown of miR-1-3p by an inhibitor produced 67-77% phenotypic females" in the mansucript.

(6) The experiment described in the paragraph starting on line 231 using a means (the white/brown Y-linked pupa marker) to establish chromosomal sex reversals seems clearly adequate to establish the relevant conclusions. Hence there would seem to be no reason to include the other, more indirect, presumably preliminary experiment (the paragraph beginning at 214 and described in Fig. 6).
Answer: Thank you for your kind suggestions. We think that the wild *Bactrocera dorsalis* strain is important and irreplaceable to test firstly the role of miR-1-3p in determining male sex in this strain because it has no genetic modification. In this study, the *white/brown pupae* sexing strain was further used to definitively determine the chromosomal sex of the mutant phenotypic females because of the Y-linked markers. In this strain, females homozygous for the recessive *white pupae* (*wp*$^-$) mutation develop as non-pigmented white pupae, whereas males, having a wild type *wp* allele (*wp*$^+$) translocated to the Y chromosome, develop as normally pigmented brown pupae. We think that the sex reversal results from these two strains would be more persuasive to verify the conclusion that miR-1-3p is sufficient to initiate male development in *B. dorsalis*.

(7) While *tra* has (finally) been found to be is present in a number of higher insects and to be involved in their sex determination, it is misleading (line 272) to characterize it as being "widely conserved" since it evolves so rapidly as to make the orthologs very difficult to identify in even closely related species.
Answer: Thank you for your kind suggestions. We have changed "widely conserved" into "functional conserved" since *tra* gene played important role in sex determination as the key switch gene in different species.

(8) Line 295: It is not necessary to review the functions of various miRNAs. Suffice it to say what is in line 294. Nobody doubts miRNAs are functionally important -- these other functions are irrelevant to the current paper.
Answer: We have removed the description of functions of various miRNAs as you suggested. Thank you.

(9) Fig 3: why refer to "expression levels" rather than simply "levels"?
Answer: We have changed "expression levels" into "levels" as you suggested. Thank you.

(10) Fig 5: Line 753. The figure legend should clearly state WHAT was injected when injections are first mentioned, not first in the second part of the figure.
Answer: We have added the material which injected into the embryo. Thank you for your kind suggestions.

Fig. 6 (although this figure and its associated text is unnecessary; Fig. 7 suffices). Panel A is unnecessary -- the mode of action of CRISPR/Cas9 is well known. The protospacer and PAM sequences targeted should simply be included in M&M. If this figure does end up in the final manuscript, the authors must indicate that the individuals in red are the PRESUMED feminized males based on an expectation of 1:1 sex ratio (actually it is unclear why the authors didn't estimate 25 rather than 24, since only 25 would have given a 1:1 sex ratio; moreover the figure would be more clear if the estimated number of transformed chromosomal males was indicated IN the red bar, with the number of estimated TRUE females indicated IN the white bar).
Answer: We have deleted Panel A and removed the protospacer and PAM sequences targeted into method and material. The individuals in red are the females with the mutant in miR-1-3p target sites. As we were not able to determine the XY and XX genotype females, we can presume the individuals in red are feminized males based on an expectation of 1:1 sex ratio. We have added more description that the individuals in red bar with the mutant in miR-1-3p target sites are the presumed feminized XY chromosomal females based on an expectation of 1:1 sex ratio and the number of estimated true XX chromosomal females indicated in the white bar as you suggested, thank you.

Reviewer #2 (Remarks to the Author):

Sex determination mechanisms are bewilderingly diverse and we are only at the beginning of uncovering the various genes involved. Sex is typically determined by a cascade of genes that is conserved at the bottom but undergoes frequent turnover at the top (primary signals). This is particularly true for the male-determining genes in insects. Four such genes have been described in the last few years from flies and mosquitoes and they are all non-homologous. Intriguingly, although they all induce the splicing of *transformer* and *doublesex* in the embryonic stage into the male mode, they may do so in different ways. Peng et al report the identification of a paternally inherited micro-RNA that functions as an (intermediate) male determiner. Although it is not the primary signal (Y-linked M factor) it identifies yet another type of regulation of sex is determination in dipteran insects. This is an interesting finding that adds to our knowledge of the complexity and evolutionary lability of the sex determination process.

1. The presented functional analyses with knock-down and induction studies are sound and the results are well-presented. Nevertheless, there are some concerns that need to be addressed, or at least be acknowledged by the authors. Why was the focus on embryonic stages 6-9 hours? Why did the authors not consider identifying the M factor in this study? They appear to have all the material (embryonic transcripts) so why not looking at earlier embryonic timepoints?
Answer: In this study, we focused on the role of small RNA in regulation of sex determination in *Bactrocera dorsalis*, and sequenced the small RNA transcriptome in

the early embryo sex determination key stages (5-7 hours) to determine whether small RNA regulate sex determination in *Bactrocera dorsalis* as that in *Bombyx mori*, in which a single female-specific piRNA is the primary determiner of sex (Kiuchi et al. 2014). We have detected relative levels of miR-1-3p in all embryonic stages 0-9 hours, including mixed embryo at 0,1,2,3,4 and 5 hpo (because female can not be separated from male embryo) (see supplemental Fig 7), single female and male embryo at 6, 7, 8 and 9 hpo (Fig 4 C). It is very pleased for us to find a male-biased microRNA, miR-1-3p, which is required for male sex determination in *Bactrocera dorsalis*. Unfortunatly, miR-1-3p is an autosomal-derived male-biased microRNA, not the primary signal (Y-linked M factor). Our small RNA sequencing data is not enough to identify such Y-linked M factor in *B. dorsalis*. It is a good suggestion and we will sequence the genomes of males and females, the transcriptome of early embryos from *B. dorsalis* to identify the Y chromosome specific gene or M factor by using the CQ method as described by Hall et al., (2015) in the future. Thank you for your kind suggestion.

2. The fact that the miR-1-3p is expressed in both males and females rightfully led the authors to conclude that it cannot be Y-linked. However, the conclusion that it is autosomal (e.g. line 31) appears premature. Can they exclude that it is X-linked? Why did they not attempt to identify it in the genome sequence (https://www.ncbi.nlm.nih.gov/assembly/GCF_000789215.1)? If this was not possible, they should at least mention this in the text. Can they exclude the existence of multiple copies, one of which is on the Y, and transmitted via sperm to eggs?
Answer: Thank you for your kind suggestions. The miRNA sequencing data showed that the genome ID of miR-1-3p is JFBF01001330.1 and transcribed from autosome (Line 135-139), not X-linked. The PCR amplification of miR-1-3p from genomic DNA isolated from female and male adult showed that miR-1-3p is not Y-linked (Line 209-211). The miRNA sequencing data which aligned to the whole genome sequence (WGS) of *B. dorsalis* indicated that miR-1-3p has only one copy on the location of JFBF01001330.1. Thank you.

3. The interpretation of the data also requires consideration. It is shown that miR-1-3p is expressed more in embryos that were assigned to be XY because these embryos express male *tra* mRNA. In principle this could be caused by this *tra* splicing. In XX individuals (assigned XX because of the presence of female *tra* mRNA) there is less miR-1-3p, that, in principle, could be caused by TRA. So if miR-1-3p lowers *tra* transcription, it needs to be shown that *tra* expression in males is lower than in females. This may be reflected in the injection experiments, but it should be mentioned. The action of miR-1-3p is then likely stabilization. The target sites described in 133-135 (UTR of a gene?) do not reveal if the target is male or female specific (probably not specific).
Answer: Our results lead us to propose a model for the sex determination pathway in *B. dorsalis* (Fig. 10): the expression of miR-1-3p is activated in early embryogenesis by a Y-linked primary signal in male embryos, potentially the putative male

determining factor (M factor) and miR-1-3p has a more direct intermediate role in inhibiting maternal *Bdtra* expression. So less miR-1-3p in XX individuals was due to the lack of Y chromosome, not caused by TRA. Anywhere, we have detected the expression of *Bdtra* in male and female embryo in 6, 7, 8 and 9 hpo (Figure 4). The results showed that the expression of female-specific *Bdtra* in male embryo is lower than in female embryo in 6 hpo embryo and female-specific *Bdtra* has degraded from 7 hpo to 9 hpo in male embryo (Figure 4A and 4B). The target site in the 3’ UTR of *Bdtra* gene is not male or female specific. The function of *Bdtra* gene in sex determination has been verified by the embryonic RNAi in our previous publication, which silencing *Bdtra* expression can lead to the masculinization (Peng et al. 2015).

Why not CRISPR the target sites of *tra*? Is the maternally provided *tra* mRNA also degraded?

Answer: Thank you for your kind suggestion. We have tried to CRISPR the target sites of *tra* as follow. To design *Bdtra* 3’ UTR specific sgRNA, all target regions have been searched with the protospacer-adjacent motifs (PAMs) of the recognition site for *Streptococcus pyogenes* Cas9 (NGG) as possible as we can find in such limited *Bdtra* 3’ UTR short sequence. Only two NGG sites were found in the 3’ UTR of *Bdtra* gene using on-line tools (following Figure 1 in this rebuttal letter). We have synthesized these two *Bdtra* 3’ UTR sgRNAs (following Figure 2 in this rebuttal letter), and the activity of these two sgRNAs was detected *in vitro*. Unfortunately, the results showed that sgRNA 1 and sgRNA 2 could not cleave the target site with the Cas9 enzyme (following Figure 3 in this rebuttal letter), which indicated that these two sgRNAs were non-activity. Thus, there is no way to knockout the target sites of *tra* using the CRISPR/Cas9 system based on such limited short sequence because there is not activity sgRNAs. As an alternative method, we have injected the miR-1-3p mutant agomir/antagomir which contained a mutation in the putative binding site into the embryo. The results showed that the female to male ratio of miR-1-3p mutant agomir/antagomir injected embryo was approximately 1:1, suggesting that miR-1-3p mutant agomir/antagomir is not effective. Those results have been added into revised mansucript (Line 219-220). In addition, the maternally provided *Bdtra* mRNA is also degraded.

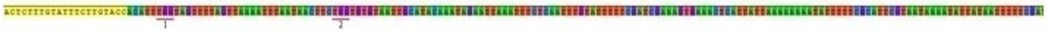

Figure 1. The schematic diagram of *Bdtra* 3’ UTR sequence. The target sites of miR-1-3p is marked in yellow. The number of 1 and 2 mean the “NGG” site of two *Bdtra* 3’ UTR sgRNA

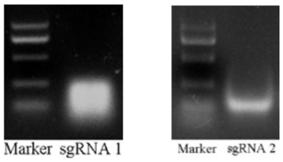

Figure 2. The illustration of two *Bdtra* 3’ UTR sgRNA

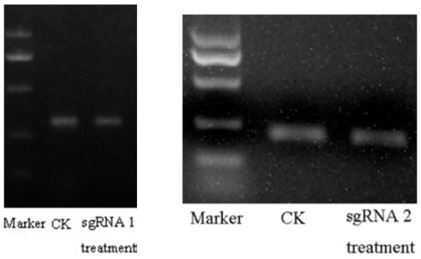

Figure 3. Activity detection of two *Bdtra* 3’ UTR sgRNA

4. The introduction lacks coherence and the different paragraphs are unbalanced. The first paragraph starts with mentioning the diversity of sex determination mechanisms but then strongly focuses on details of the *Drosophila* sex determination cascade, which is just one example, and an exceptional one, that is not related much to the study presented as it does not have an M factor. The second paragraph lacks a closing sentence. Focussing the introduction on species that do have M would be more appropriate. What was the purpose of the study (cf line 92)? To determine the crucial sex determining window, but then the miRNA was accidentally hit upon? If so, present it that way.
Answer: We have revised the introduction as you suggested, thank you. The purpose of the study is to identify the male determined factor in *Bactrocera dorsalis*. In this study, we focused on the role of small RNA in regulation of sex determination in *Bactrocera dorsalis*, and sequenced the small RNA transcriptome in the early embryo sex determination key stages (5-7 hours) to determine whether small RNA regulate sex determination in *Bactrocera dorsalis* as done in *Bombyx mori*, in which a single female-specific piRNA is the primary determiner of sex in previous study (Kiuchi et al. 2014).

There are a number of inaccurate or in appropriate statements throughout the manuscript:
5. In the title and rest of the text the term “male-biased” is used. The term is not entirely correct as the miRNA works before sex determination, i.e. it is not male-biased but male-inducing. The term is also confusing with other genomic studies that compare gene expression levels between the sexes.
Answer: We have deleted “male-biased” in the title and the rest of the text in our manuscript to avoid the confusing meanings. Thank you for your kind suggestion.

6. Why calling the M-factor “MDF” as M-factor is typically used in the literature?
Answer: We have changed the “MDF” into “M-factor”. Thank you for your kind suggestion.

7. The piRNAs do not act on *tra*, and are therefore also inappropriately placed in the intro. In addition they are feminizing not masculinizing.
Answer: You are right, the sex chromosomes system in *Bombyx mori* is different from that in *B. dorsalis*. The silkworm *B. mori* uses a WZ sex determination system, no *tra* ortholog has been identified in *B. mori*, the W-derived piRNA acts on the *Masc* gene

which an upstream gene before *doublesex* gene. *B. dorsalis* uses a XY sex determination system with *tra*. But in this study, we focused on the role of small RNA, which include miRNAs and piRNAs, in regulation of sex determination in *B. dorsalis* by sequencing the small RNA transcriptome in the early embryo sex determination key stages (5-7 hours) to determine whether small RNA regulate sex determination in *B. dorsalis* as done in *B. mori* (Kiuchi et al. 2014). So piRNAs were placed in the introduction, in order to reflect the diversity of sex determination mechanisms and the role of small RNA in sex determination in insects. Thank you for your kind suggestion.

8. Throughout the paper "splicing of *tra*" or any other gene is used, when the splicing of the pre-mRNA (primary transcript) is meant.
Answer: We have corrected this issue (such as changing "splicing of *tra*" into "splicing of *tra* pre-mRNA). Thank you for your kind suggestion.

For more specific terminology see below under minor comments
There are a number of methodological issues:
9. The first experiment described in lines 104-115 is not included in the M&M or is it line 441-442 (which would be in the wrong spot of the M&M)? Why only take 5-7 h for this screen? Taking earlier time points would have eliminated the uncertainty whether the 5 h amplification is still maternal provision of *tra* mRNA.
Answer: The first experiment described in lines 104-115 is in line 441-442 and is in the wrong spot of the M&M. We have removed this sentence into the beginning of sample preparation section. We have checked the female and male transcripts of *Bdtra* from 0-10 hpo at early embryogenesis, not only take 5-7 hpo. The appearance of *Bdtra-m* at 6 hpo embryo and degradation of *Bdtra-f* at 7 hpo embryo suggested that the key stage determining the embryo male sex determination is during 5, 6 and 7 hpo. The maternal provision of *tra* mRNA is existed from 0 hpo to 6 hpo (XX and XY embryo), but degraded in 7 hpo XY embryo. Thank you for your kind suggestion.

10. N=1 per category is tricky for mixed-sex small RNA datasets, N=3 per non-mixed category is on the lower acceptable end for quantitative analyses according to the LGTC.
Answer: Although the mixed-sex small RNA datasets was constructed once per category, the quantitative analyses were performed at least three times in all other experiments in the manuscript. Thank you for your kind suggestion.

11. Supplementary table 2 shows the normalized abundance of particular miRNAs and this is the same data that was used for the tests (line 130) that showed that these increased significantly at hpo 7 compared to 5 and 6. How does this work? As in many cases the hpo 7 value is lower than the other two categories. The amount of reads after filtering is not noticeably different between the 3 libraries.
Answer: Yes, the normalized abundance of miRNAs in 7 hpo is lower than the other two categories in many cases. We have deleted the sentence "increased significantly at

7 hpo compared to 5 and 6 hpo" in line 130. 65 miRNAs at 5, 6 and 7 hpo were differentially expressed was confirmed by the P value (chi square) which is lower than 0.05. Thank you for your kind suggestion.

12. Why only look at miRNA when the only other known small RNA sex determination factor is a piRNA in *Bombyx* ?

Answer: In the silkworm *Bombyx mori*, female-enriched PIWI-interacting RNAs (piRNAs) are the only known transcripts that are produced from the sex-determining region of the W chromosome (Kawaoka et al. 2011). While the major component of tephritid Y chromosomes is repeat-rich sequences, not is piRNA. We sequenced the small RNA library which including the miRNA and piRNA, but the results showed that all component of the libraries are miRNA, not piRNA in *B. dorsalis*, and found that the miRNA is required for male sex determination in *B. dorsalis*. Thank you for your kind suggestion.

13. miR-276a-3p and miR12-5p were suggested from the luciferase assay to target *tra*, yet these fell of the candidate list at the *in vivo* test. Could they still be involved, but in example earlier than the measured 10 h post-injection time point? Have they been tested in single-sex samples to examine whether they are male-specific?

Answer: To test the interaction between miR-276a-3p, miR-12-5p and *Bdtra* in earlier than the measured 10 h post-injection time point, we injected newly laid embryos with miR-276a-3p and miR-12-5p agomirs/antagomirs. The results showed that the expression level of *Bdtra* was not affected by miR-276a-3p and miR-12-5p agomir and antagomir treatments in 6 h post-injection embryos (Figure 4 in this rebuttal letter). We have checked the expression of miR-276a-3p and miR12-5p in single female/male embryo at 6, 7, 8, 9 hpo. The results showed that miR-276a-3p and miR12-5p are both not male-specific miRNAs (Figure 5 in this rebuttal letter). Thank you for your kind suggestion.

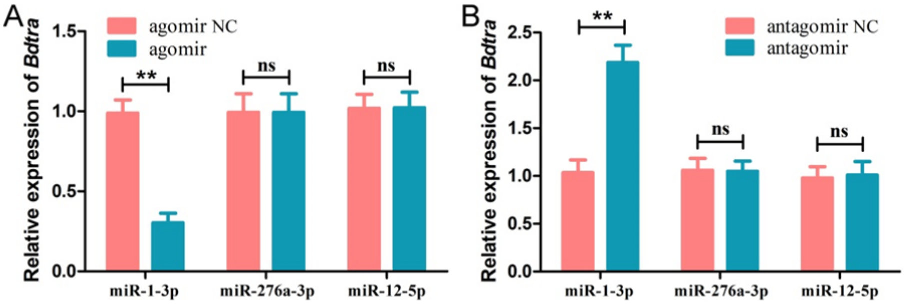

Figure 4. Relative levels of *Bdtra* in 6 h embryos injected with miR-1-3p, miR-12-5p and miR-276a-3p agomir (A) and antagomir (B)

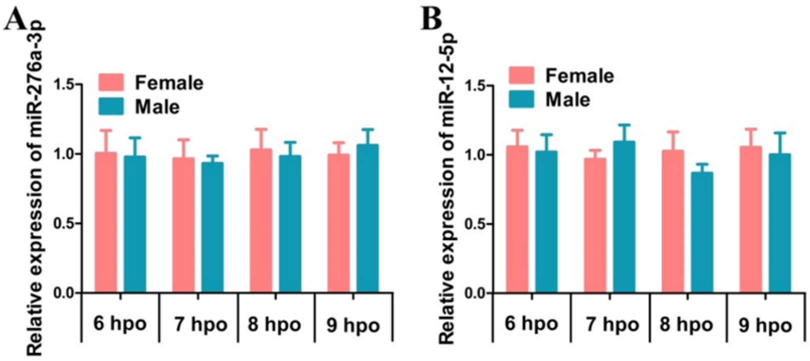

Figure 5. Relative levels of miR-276a-3p (A) and miR-12-5p (B) in single female and male embryo at 6, 7, 8 and 9 hpo

14. Line 167: where does the identification of *Dicer-1* in *B. dorsalis* come from? Predicted annotation from genome assembly? Why are the different sample categories of supplementary figure 6A not mentioned in the M&M?

Answer: Yes, we identified *Dicer-1* gene from *B. dorsalis* genome (https://www.ncbi.nlm.nih.gov/assembly/GCF_000789215.1). The gene ID is LOC105232610. We have added the information of different sample categories of supplementary figure 6A in the M&M. Thank you for your kind suggestion.

Minor comments

15. Line 23: what is "male determination"?

Answer: Sex determination is an essential developmental program, for which insects have evolved diverse mechanisms including male determination by highly diverse Y chromosome-linked male determining factors (M factors) (Criscione et al., *eLife* 2016; Hall et al., *Science* 2015; Krzywinska et al., *Science* 2016; Sharma et al., *Science* 2017). Thank you.

16. Line 27: what is "a male-biased RNA"?

Answer: A male-biased RNA means the expression level of RNA is highly expressed in males when compared to the expression level in females. Thank you.

17. Line 37: what is "and intermediate male determiner"?

Answer: The intermediate male determiner means the factor between the primary male determining factor and the key sex determination *tra* gene. Thank you.

18. Lines 59-60: this is not entirely correctly phrased: *Mdmd* prevents the zygotic activation of the *tra* loop, but not activation of *tra* itself.

Answer: We have corrected this mistake. Thank you for your kind suggestion.

19. Lines 53-61: The recently discovered M in Musca is inaccurately described. Mentioning the CWC22 paralog would be appropriate. M in Musca is not necessarily

Y-linked. The word “similarly” in line 61 is inappropriate.
Answer: We have added the “*CWC22* paralog” into the describing of male determiner in *M. domestica* as you suggested. We have deleted the word “similarly”, thank you.

20. Lines 60-61: remove once “splicing”, moreover, genes are not spliced, the transcripts are.
Answer: We have revised this sentence into “resulting in the default male-specific splicing of *Mdtra* and *Mddsx* pre-mRNAs leading to male differentiation” as you suggested. Thank you so much.

21. Line 65: “none have yet to be identified” means that they have all been identified!
Answer: No, I am sorry not to describe clearly. Here we want to say that “Y-linked M-factors have been inferred in dipteran insects, but have not yet been identified in tephritid fruit flies including the oriental fruit fly”. We have corrected this mistake. Thank you so much.

22. Lines 70-76: difficult to read, split up into two sentences. This sentence also contains inaccuracies
Answer: We have revised this sentence into “As maternal *tra* transcripts initiate a positive autoregulatory feedback loop in the early zygote, continuous female-specific functional Tra protein is provided and, thus, acts as a cellular memory, maintaining the female-determining signal[15]. Thank you for your kind suggestion.

23. Line 80: SIT is not previously introduced and not well integrated in the text here
Answer: We have started the SIT information as a new paragraph. Thank you for your kind suggestion.

24. Line 86: should “downstream factors” not be “upstream factors” in the cascade?
Answer: We have corrected “downstream factors” into “upstream factors” as you suggested. Thank you.

25. Line 94: what is meant with “a male-biased expression”, better: is expressed only in male embryos? Also “Line 195-196: is male-biased expressed in the male embryo”
Answer: Male-biased expression means the expression level of miRNA is highly expressed in male embryo compared to the expression level in female embryo. Male-biased is not expressed only in male embryos. We have revised the sentence “has a male-biased expression” into “induces male sex determination by suppressing *Bdtra* expression” and “The expression patter of miR-1-3p gene in male and female *B. dorsalis* during early embryonic development showed that miR-1-3p is male-biased expressed in the male embryo” into “the relative expression levels of miR-1-3p in single male embryos were significantly higher than in single female embryos”. Thank you.

26. Lines 104-114: Looks like the sex of the embryo is determined by assessing the

tra splice forms. It is a bit misleading to say that male splice forms occur in XY embryo's, if the labelling of XY is derived from the identification of such a splice form.

Answer: Based on the detection of only *Bdtra-m* in a single XY embryo, which is the major characteristic of sex-specific splicing of *Bdtra* pre-mRNA, we identified the sex of individual female and male embryos. Thank you.

27. Line 116: why do the authors expect such RNAs to be present?

Answer: In previous study, small RNAs have been found to have many functions in insects, especially, a single female-specific piRNA has been found to be the primary determiner of sex in the silkworm moth, *Bombyx mori* (Kiuchi et al. 2014). This female-specific piRNA represses transcription of *Masculinizer* (*Masc*) which is a key gene in the sex determination cascade. So we want to know the role of small RNA in regulation of sex determination in *Bactrocera dorsalis*. That is why we expect such RNAs to be present. Thank you.

28. Line 131: How do you know the miRNAs are autosomal?

Answer: Thank you for your kind suggestions. The miRNA sequencing data showed that the genome ID of miR-1-3p is JFBF01001330.1 and transcribed from autosome (Line 135-139), not X-linked. The PCR amplification of miR-1-3p from genomic DNA isolated from female and male adult showed that miR-1-3p is not Y-linked (Line 209-211). Thank you.

29. Line 192-193: check phrasing (English)

Answer: We have revised this sentence into "Based on the detection of only *Bdtra-m* in a single XY embryo, which is the major characteristic of sex-specific splicing of *Bdtra* pre-mRNA, we identified the sex of individual female and male embryos at 6, 7, 8 and 9 hpo". Thank you for your kind suggestion.

30. Line 194: patter = pattern

Answer: We have corrected this mistake. Thank you so much.

31. Line 272-273: replace "cases" by "orders" and delete last part of sentence

Answer: We have replaced "cases" by "orders" and deleted last part of sentence as you suggested. Thank you.

32. Lines 290-297: these seem results that are not described in the result-section itself. Here it just distracts from the discussion on candidate primary signals in sex determination.

Answer: We have deleted all these sentences. Thank you for your kind suggestion.

33. Line 296: delete "which"

Answer: It is a very kind suggestion. We have deleted "which", thank you.

34. Line 322 and throughout the manuscript: The use of “while”. Strictly speaking, “while” is used to refer to time, so “whereas” would be the better term.
Answer: We have changed the “while” into “whereas” as you suggested. We have checked and corrected the use of “while” throughout the manuscript. Thank you so much.

35. Line 337: the term “M factor” is introduced here for the first time, needs explanation
Answer: We have added the explanation of “M factor” in this sentence. Thank you for your kind suggestion.

36. Line 356 hints to the use of this miRNA in other tephritid species. Is it known to be conserved in other model systems of this family?
Answer: Our study is the first report of a direct role for this miRNA in determining the male sex in early embryogenes of insects. It needs further confirmation whether it is conserved in other model systems of this family. We have deleted the sentence “and perhaps other tephritid species”. Thank you.

37. Line 366-367: why is the sex ratio of the white pupae strain different from 1:1?
Answer: The strain normally generates a 1:3 female to male ratio as described in previous study (Mccombs et al., 1995). Thank you.

38. Line 500: “all experiments were repeated at least 3 times” The small RNA sequencing was presumably not. Are the other sample sizes mentioned in the manuscript the cumulative number of all these experiments, or of the larger main experiment (versus non-described pilot studies)?
Answer: The small RNA sequencing was not repeated three times and only one small RNA library was constructed from each time point. The other sample sizes mentioned in the manuscript are the number of each treatment, not the cumulative number of all these experiments. We have changed the words into “all experiments were repeated at least 3 times except for the small RNA sequencing”. Thank you.

Reviewer #3 (Remarks to the Author):

This manuscript by Peng et al. describes a role for miR-1-3p in male sex determination in the oriental fruit fly, *Bactrocera dorsalis*. Key experiments are presented at the end of the paper showing that CRISPR-based mutation of miR-1 leads to sex reversal of XY individuals and, conversely, that miR-1 agomir injections leads to sex reversal of XX individuals. These key experiments are complemented by expression and molecular analyses supporting the regulation *Bdtra* by miR-1. Overall, the experiments are technically sound and properly controlled, the results are clearly presented and appropriately interpreted and, for the most part, the text is well written. However, there are a few concerns, outlined below, that should be addressed to strengthen the manuscript.

(1) The conclusion that sex determination occurs between 6 and 7 hpo does not seem supported by the data. Sex specific splicing is detected at 6 hpo, indicating that the process of sex determination has already begun at this time.

Answer: You are right, we describe incorrectly. The sex-specific splicing of *Bdtra* in early embryo show that there is no male-specific *Bdtra* splicing at 5 hpo and male-specific *Bdtra* transcript was first detectable at 6 hpo. These data indicated that sex determination occurs between 5 and 6 hpo. We have revised the conclusion. Thank you for your kind suggestion.

(2) miR-1 expression level should be shown prior to 6 hpo in Fig 4C. Presumably there should be a sexually dimorphic switch in miR-1 expression that leads to sex-specific splicing of *tra* at 6 hpo. If miR-1 expression is the same at 5 hpo (or 4 hpo) as it is at 6 hpo, then it cannot be the presumed trigger that causes the switch in *tra* splicing.

Answer: We have detected relative levels of miR-1-3p in all embryonic stages 0-9 hours, including mixed embryo at 0, 1, 2, 3, 4 and 5 hpo (because female embryo could not be separated from male embryo) (Supplemental Fig 7), single female and male embryo at 6, 7, 8 and 9 hpo (Fig 4C). The results show that the expression level of miR-1 increased from 0 hpo to 6 hpo, especially a significant increased from 5 hpo to 6 hpo, indicating that there is a sexually dimorphic switch in miR-1 expression that leads to sex-specific splicing of *tra* at 6 hpo. Thank you for your kind suggestion.

(3) Ideally, the authors would support the effect of miR-1 on *tra* with Western blots of Tra protein levels in wild type and miR-1 mutant males and females.

Answer: We have checked the BdTRA protein levels using Western blots in normal and miR-1 mutant male and female, and these results have been incorporated into the body of the revised manuscript (Lines 278-282) (Fig. 9A and 9B). The Western blots results support the regulation effect of miR-1 on *tra* expression and show that BdTRA protein levels were normally detected in the mutant males (feminized individuals from males) as in normal and mutant females while they could hardly be detected in normal males (Fig. 9A and 9B). Thank you for your kind suggestion.

(4) The authors should show that injection of a miR-1-insensitive version of the *tra* transcript containing a mutation in the putative miR-1 binding site leads to sex reversal of XY individuals. This does not seem like a difficult experiment, and would provide significant additional support for the model. In addition, the authors could consider analyzing the effects of CRISPR-based mutations in the miR-1 binding site of the *tra* 3'UTR.

Answer: As you suggested, we have added an experiment to inject the miR-1-3p mutant agomir/antagomir which contained a mutation in the putative binding site into the embryo. The results showed that the female to male ratio of miR-1-3p mutant agomir/antagomir injected embryo was approximately 1:1, suggesting that miR-1-3p mutant agomir/antagomir is not effective. Those results have been added into the

manuscript (Line 220-221, Supplementary Table 5).

About the effects of CRISPR-based mutations in the miR-1 binding site of the *tra* 3'UTR, we firstly searched all target regions of the miR-1 binding site with the protospacer-adjacent motifs (PAMs) of the recognition site for *Streptococcus pyogenes* Cas9 (NGG) to design *Bdtra* 3' UTR specific sgRNA as possible as we can in such limited *Bdtra* 3' UTR short sequence. Only two NGG sites were found in the 3' UTR of *Bdtra* gene using on-line tools in such limited *Bdtra* 3' UTR short sequence (following Figure 1 in this rebuttal letter). Then synthesized these two *Bdtra* 3' UTR sgRNAs (following Figure 2 in this rebuttal letter) and the activity of these two sgRNAs was detected *in vitro*. Unfortunatly, the results showed that sgRNA 1 and sgRNA 2 could not cleave the target site with the Cas9 enzyme (following Figure 3 in this rebuttal letter), which indicated that these two sgRNAs were non-activity. Thus, there is no way to knockout the target sites of *tra* using the CRISPR/Cas9 system based on such limited short sequence because there is not activity sgRNAs in such limited *Bdtra* 3' UTR short sequence as answer to Reviewer #2's comment 3 above. Anywhere, the above result that miR-1-3p mutant agomir/antagomir is not effective (Supplementary Table 5) confirm this point as an alternative method. Thank you for your kind suggestion.

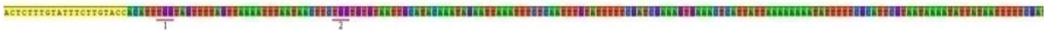

Figure 1. The schematic diagram of *Bdtra* 3' UTR sequence. The target sites of miR-1-3p is marked in yellow. The number of 1 and 2 mean the "NGG" site of two *Bdtra* 3' UTR sgRNA

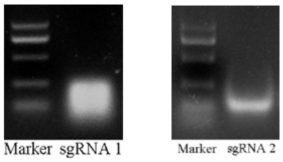

Figure 2. The illustration of two *Bdtra* 3' UTR sgRNA

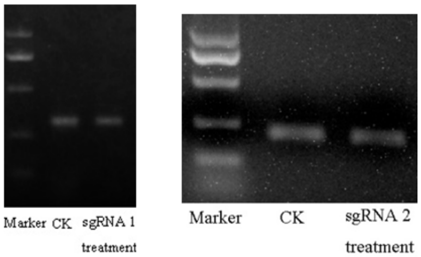

Figure 3. Activity detection of two *Bdtra* 3' UTR sgRNA

(5) One issue that needs to more fully addressed is how the authors reconcile the sexually dimorphic role of miR-1 in *Bactrocera dorsalis* with its presumably non-dimorphic role in muscle. Is the sexually dimorphic expression that the authors describe in Fig 4C specific to early embryogenesis? Is miR-1 expressed at equivalent levels in males and females at later stages? The authors should show a broader temporal profile of miR-1 expression to address these issues.

Answer: In our study, we only focused and found that miR-1 plays pivotal role in the male sex determination in *Bactrocera dorsalis*, but did not have sufficient evidences to determine whether miR-1 is involved in non-dimorphic role in muscle based on our research data. As one miRNA can participate in different process by regulating different genes, we just focused on the role of miR-1 in the male sex determination. The sexually dimorphic expression that described in Fig 4C is specific to early embryogenesis. miR-1 is not expressed at equivalent levels in males and females at later stages. We have added a broader temporal profile of miR-1 expression in the immature (IM), middle-aged (MA) and fully mature (FM) female and male adults of *B. dorsalis* (Supplemental Fig. 7B). These results showed that miR-1 has sexually dimorphic expression also during adult development stages (Line 207-209). Thank you for your kind suggestion.

(6) I'm not sure that it is appropriate to conclude that miR-8-3p, miR-10-5p, miR-308-3pm, miR-10-3p and miR-305-5p don't regulate *Bdtra* in human cells (lines 148-149). Isn't it possible that orthologues of these miRNAs are endogenously expressed in HEK293T, leading to luciferase repression in the absence of the mimics?
Answer: Firstly, miR-8-3p, miR-10-5p, miR-308-3pm, miR-10-3p and miR-305-5p are insect species-specific miRNAs, so there are no these endogenous miRNAs in human HEK293T cell lines. Furthermore, in the case that one miRNA is endogenously expressed in the cell lines, the concentration level of this endogenous miRNA is extremely lower than that obtained by transfecting this miRNA mimics into the cell lines. So, the transfection of this miRNA mimics should also be able to induce a further luciferase reduction of the target gene. So Dual luciferase reporter (DLR) assay is a technique commonly used to check the relationship between the miRNA and the target gene site (Ling et al., 2017; Zhang et al., 2016). Thank you.

(7) There are some issues with the writing. Awkward sentences include those on lines 71-76, lines 107-109, and lines 191-194.
Answer: We have revised the sentence on lines 71-76 into "In contrast to *Drosophila*, the *tra* gene in *B. dorsalis* as well as other dipteran, hymenopteran and coleopteran species[10, 15-20], is able to autoregulate its female-specific splicing, analogous to the *Drosophila Sxl* gene. As maternal *tra* transcripts initiate a positive autoregulatory feedback loop in the early zygote, continuous female-specific functional Tra protein is provided and, thus, acts as a cellular memory, maintaining the female-determining signal[15]", revised the sentence on lines 107-109 into "This was achieved by analyzing *Bdtra* sex-specific splicing isoforms in individual embryos at distinct hours post-oviposition (hpo) time points, thereby distinguishing the chromosomal sex of the embryos and their sexual fate" and revised the sentence on lines 191-194 into "Based on the detection of only *Bdtra-m* in a single XY embryo, which is the major characteristic of sex-specific splicing of *Bdtra* pre-mRNA, we identified the sex of individual female and male embryos at 6, 7, 8 and 9 hpo (Fig. 4A and 4B)". Thank you for your kind suggestion.

Minor Comments

(1) Line 95: miR-1 acts to regulate *Bdtra* transcription? The authors haven't shown this, but have shown an effect on *Bdtra* transcript level and splicing.
Answer: miR-1 acts to regulate the expression level of *Bdtra* by degradation and not modulate transcription. We have corrected this issue, thank you.

References:

Criscione F, Qi Y, Tu ZJ. *GUY1* confers complete female lethality and is a strong candidate for a male-determining factor in *Anopheles stephensi*. *eLife* 5, (2016).

Gabrieli P, *et al.* Sex and the single embryo: early deveopment in the Mediterranean fruit fly, *Ceratitis capitata*. *BMC Dev. Biol.* **10**, 12-12 (2010).

Hall AB, *et al.* A male-determining factor in the mosquito *Aedes aegypti*. *Science* **348**, 1268-1270 (2015).

Kawaoka, S. et al. The silkworm W chromosome is a source of female-enriched piRNAs. *RNA* **17**, 2144–2151 (2011).

Kiuchi T, *et al.* A single female-specific piRNA is the primary determiner of sex in the silkworm. *Nature* **509**, 633-636 (2014).

Krzywinska E, Dennison NJ, Lycett GJ, Krzywinski J. A maleness gene in the malaria mosquito *Anopheles gambiae*. *Science* **353**, 67-69 (2016).

Ling L, Kokoza V, Zhang C, Aksoy E, Raikhel A. MicroRNA-277 targets *insulin-like peptides 7* and *8* to control lipid metabolism and reproduction in *Aedes aegypti* mosquitoes. *Proc. Natl. Acad. Sci. USA* **114**, 8017-8024 (2017).

Mccombs SD, Saul SH. Translocation-based genetic sexing system for the oriental fruit fly (Diptera: Tephritidae) based on pupal color dimorphism. *Ann. Entomol. Soc. Am.* **88**, 695-698 (1995).

Morrow JL, Riegler M, Frommer M, Shearman DCA. Expression patterns of sex-determination genes in single male and female embryos of two *Bactrocera* fruit fly species during early development. *Insect Mol. Biol.* **23**, 754-767 (2014).

Peng W, Zheng W, Handler AM, Zhang H. The role of the *transformer* gene in sex determination and reproduction in the tephritid fruit fly, *Bactrocera dorsalis* (Hendel). *Genetica* **143**, 717-727 (2015).

Sharma A, et al. Male sex in houseflies is determined by *Mdmd*, a paralog of the generic splice factor gene *CWC22*. *Science* **356**, 642-645 (2017).

Zhang Y, *et al.* microRNA-309 targets the Homeobox gene *SIX4* and controls ovarian development in the mosquito *Aedes aegypti*. *Proc. Natl. Acad. Sci. USA* **113**, 4828-4836 (2016).

REVIEWERS' COMMENTS:

Reviewer #1 (Remarks to the Author):

The authors have responded remarkably constructively to all the many concerns of the three reviewers. Hence the manuscript is much improved in both experimental content, logical presentation, and language style and is publishable in its present form. (line 307: functionalLY conserved)

Thomas Cline

Reviewer #2 (Remarks to the Author):

In this revised manuscript, Peng et al report the identification of a paternally inherited micro-RNA that plays a role in sex determination of the fruit fly Bactrocera dorsalis. This is another valuable addition to the bewildering diversity in gene regulation of sex is determination in insects. My main concerns have been well addressed:

- The identified micro RNA is clearly involved in sex determination and earlier embryonic time points will be investigated for the primary signal at a later stage
- miR-1-3p is autosomal based on the genome sequence
- regulation on miR-1-3p in males and females is addressed
- the introduction is now more comprehensively written
- ambiguous terminology has been improved
- methodological issues have been addressed

Reviewer #3 (Remarks to the Author):

The authors have fully addressed my previous comments in this revised manuscript.

REVIEWERS' COMMENTS:

Reviewer #1 (Remarks to the Author):

The authors have responded remarkably constructively to all the many concerns of the three reviewers. Hence the manuscript is much improved in both experimental content, logical presentation, and language style and is publishable in its present form. (line 307: functionalLY conserved)
Thomas Cline
Answer: We have revised "functional conserved" into "functionally conserved", thank you for your kind suggestions.

Reviewer #2 (Remarks to the Author):

In this revised manuscript, Peng et al report the identification of a paternally inherited micro-RNA that plays a role in sex determination of the fruit fly Bactrocera dorsalis. This is another valuable addition to the bewildering diversity in gene regulation of sex is determination in insects. My main concerns have been well addressed:
- The identified micro RNA is clearly involved in sex determination and earlier embryonic time points will be investigated for the primary signal at a later stage
- miR-1-3p is autosomal based on the genome sequence
- regulation on miR-1-3p in males and females is addressed
- the introduction is now more comprehensively written
- ambiguous terminology has been improved
- methodological issues have been addressed

Reviewer #3 (Remarks to the Author):

The authors have fully addressed my previous comments in this revised manuscript.
